# Orally Ingested Probiotic, Prebiotic, and Synbiotic Interventions as Countermeasures for Gastrointestinal Tract Infections in Nonelderly Adults: A Systematic Review and Meta-Analysis^☆^

**DOI:** 10.1016/j.advnut.2023.02.002

**Published:** 2023-02-22

**Authors:** Heather S. Fagnant, Sandra D. Isidean, Lydia Wilson, Asma S. Bukhari, Jillian T. Allen, Richard T. Agans, Dustin M. Lee, Adrienne Hatch-McChesney, Claire C. Whitney, Elaine Sullo, Chad K. Porter, J. Philip Karl

**Affiliations:** 1 US Army Research Institute of Environmental Medicine, Natick, MA, United States; 2 Naval Medical Research Center, Silver Spring, MD, United States; 3 Henry M. Jackson Foundation for the Advancement of Military Medicine, Inc., Bethesda, MD, United States; 4 The George Washington University, Washington, DC, United States; 5 Oak Ridge Institute of Science and Education, Belcamp, MD, United States; 6 U.S. Air Force School of Aerospace Medicine, Dayton, OH, United States; 7 Brooke Army Medical Center, Fort Sam Houston, TX, United States

**Keywords:** gastrointestinal illness, infectious diarrhea, travelers’ diarrhea, gut microbiome, fermentable fiber, dietary supplement

## Abstract

Meta-analyses have not examined the prophylactic use of orally ingested probiotics, prebiotics, and synbiotics for preventing gastrointestinal tract infections (GTIs) of various etiologies in adult populations, despite evidence that these gut microbiota-targeted interventions can be effective in treating certain GTIs. This systematic review and meta-analysis aimed to estimate the effects of prophylactic use of orally ingested probiotics, prebiotics, and synbiotics on GTI incidence, duration, and severity in nonelderly, nonhospitalized adults. CENTRAL, PubMed, Scopus, and Web of Science were searched through January 2022. English-language, peer-reviewed publications of randomized, placebo-controlled studies testing an orally ingested probiotic, prebiotic, or synbiotic intervention of any dose for ≥1 wk in adults who were not hospitalized, immunosuppressed, or taking antibiotics were included. Results were analyzed using random-effects meta-analyses of intention-to-treat (ITT) and complete case (CC) cohorts. Heterogeneity was explored by subgroup meta-analysis and meta-regression. The risk of bias was assessed using the Cochrane risk-of-bias 2 tool. Seventeen publications reporting 20 studies of probiotics (*n* = 16), prebiotics (*n* = 3), and synbiotics (*n* = 1) were identified (*n* > 6994 subjects). In CC and ITT analyses, risk of experiencing ≥1 GTI was reduced with probiotics (CC analysis—risk ratio: 0.86; 95% CI: 0.73, 1.01) and prebiotics (risk ratio: 0.80; 95% CI: 0.66, 0.98). No effects on GTI duration or severity were observed. Sources of heterogeneity included the study population and number of probiotic strains administered but were often unexplained, and a high risk of bias was observed for most studies. The specific effects of individual probiotic strains and prebiotic types could not be assessed owing to a lack of confirmatory studies. Findings indicated that both orally ingested probiotics and prebiotics, relative to placebo, demonstrated modest benefit for reducing GTI risk in nonelderly adults. However, results should be interpreted cautiously owing to the low number of studies, high risk of bias, and unexplained heterogeneity that may include probiotic strain-specific or prebiotic-specific effects.

This review was registered at PROSPERO as CRD42020200670.

**Statement of significance**

Previous meta-analyses on the prophylactic use of probiotics, prebiotics, or synbiotics for gastrointestinal tract infections (GTIs) in nonhospitalized adults have focused solely on travelers’ diarrhea; included antibiotic-associated diarrhea; or considered only probiotic interventions. This systematic review and meta-analysis extended that evidence base by suggesting favorable effects of both probiotic and prebiotic interventions on reducing the risk of experiencing ≥1 GTIs of various etiologies in nonelderly, nonhospitalized adults and by identifying the study environment and number of probiotic strains included in an intervention as sources of heterogeneity for some outcomes. However, analyses could not address the specific effects of different probiotic strains or prebiotic types owing to a lack of confirmatory trials, and findings require cautious interpretation owing to limitations in the available evidence, collectively highlighting a need for additional, confirmatory research.

## Introduction

Gastrointestinal tract infections (GTIs) are common and pose a significant global health burden. Caused by a wide range of bacterial, viral, and parasitic agents, GTIs are often related to poor sanitation and poverty in developing countries and seasonality, travel, foodborne transmission, and nosocomial infection in developed countries [1]. GTI symptoms often include diarrhea or dysentery [2] and are frequently self-limiting but can also lead to severe debilitation or death, especially in developing countries. Diarrhea caused by GTIs ranked among the top causes of death and disability-adjusted life-years worldwide in 2019 [3], and in the United States, foodborne illness affects an estimated 1 in 6 people annually at a cost of >$15.6 billion [4]. GTIs are also a common complaint among generally healthy travelers, military personnel, and athletes: ∼30% of military personnel and civilians traveling for >1 mo are incapacitated or unable to participate in daily activities owing to GTIs [5]; acute diarrheal illness is the most common noncombat disease among deployed United States personnel [6]; and GTIs are one of the most common causes of illness among elite athletes [7].

Therefore, preventing GTI is a global public health priority. Preventative measures include behavioral changes, such as frequent hand washing, avoiding high-risk foods and beverages, and limiting close contacts. Medical interventions, such as prophylactic antibiotics and vaccines and bismuth subsalicylate, are also available. However, in certain cases, these interventions may not be practical or accessible owing to infrastructural, economic, or cultural barriers [[8], [9], [10]], and behavioral modifications have limited efficacy for reducing GTIs among travelers in some parts of the world [11]. In addition, systemic prophylactic antibiotics are not recommended because of their potential role in antibiotic resistance development and side effects, such as perturbing the host gut microbiota [12,13], whereas medications, such as bismuth subsalicylate, also have side effects and may not be safe for all at-risk individuals [14]. Therefore, interventions for preventing GTI that are accessible, safe, and cost-effective could play an important role in reducing the burden to health care systems and improving the quality of life of individuals at high-GTI risk.

Orally ingested probiotics, prebiotics, and synbiotics are gut microbiota-targeted interventions that may help fill that role. Probiotics, found in some fermented foods and dietary supplements, introduce exogenous microbes into the gut microbiota, if only transiently, and are defined as, “live microorganisms that, when administered in adequate amounts, confer a health benefit on the host” [15]. By contrast, prebiotics, which include fermentable dietary and functional fibers, such as inulin, fructooligosaccharides (FOS) [16], and galactooligosaccharides (GOSs), nourish the commensal gut microbiota and are defined as, “substrates that are selectively utilized by host microorganisms, conferring a health benefit” [17]. Finally, synbiotics both add to and nourish the commensal microbiota and are defined as “mixtures comprising live microorganisms and substrates selectively utilized by host microorganisms that confer a health benefit on the host” [18]. Evidence suggests that these gut microbiota-targeted interventions could help combat GTI through various mechanisms, such as inhibiting and competitively, excluding pathogens, reducing colonic pH, antimicrobial action, and strengthening immune or gut barrier functions [15,19].

Recent meta-analyses of clinical studies that assessed the efficacy or effectiveness of probiotic, prebiotic, or synbiotic interventions for reducing GTI incidence, duration, or severity have predominately focused on GTI treatment [[20], [21], [22], [23], [24], [25], [26], [27], [28], [29], [30], [31], [32], [33], [34], [35]], often in pediatric populations [20,22,23,27,[29], [30], [31], [32], [33], [34]]. Fewer have examined the prophylactic use of probiotics, prebiotics, or synbiotics for reducing GTI risk in adults. Several of those reported a reduced odds or risk of *Clostridium difficile–*associated diarrhea or infection in adults and children taking prophylactic probiotics [16,36,37]. However, the generalizability of these results to free-living populations or other pathogens is uncertain given that studies included in the meta-analyses relied primarily on hospitalized patients who were often taking gut microbiota–disrupting antibiotics and who likely experienced unique risk factors. In addition, results in pediatric populations may not be broadly generalizable given that the gut microbiota and immune systems of infants and children differ from those of adults [38]. Meanwhile, meta-analyses on the prophylactic use of probiotics, prebiotics, or synbiotics on GTIs in nonhospitalized adults have focused solely on travelers’ diarrhea (TD) [39]; included antibiotic-associated diarrhea [8,35]; or considered only probiotic interventions [8,35,39]. Therefore, whether prophylactic probiotic, prebiotic, and/or synbiotic use reduces the GTI burden among healthy adults remains unclear. This systematic review and meta-analysis aimed to determine the prophylactic effects of orally ingested probiotics, prebiotics, and synbiotics on the incidence, duration, and severity of GTIs of any etiology in otherwise healthy, nonhospitalized, nonelderly adult populations. Secondary objectives were to explore potential sources of heterogeneity to include the study population and the type, dose, and duration of supplementation.

## Methods

This review was performed in accordance with the PRISMA statement [40]. The review protocol was registered on November 27, 2020 in the PROSPERO international register of systematic reviews (National Institute for Health Research, University of York, United Kingdom; https://www.crd.tork.ac.uk/prospero/; registration number CRD42020200670).

### Search strategy

The Cochrane Central Register of Controlled Trials (CENTRAL), PubMed, Scopus, and Web of Science databases were searched on 30 October 2020 without restriction regarding the date of publication. Search queries were developed in consultation with a knowledge synthesis librarian. Searches used terms and medical subject headings designed specifically for both interventions and outcomes and included a validated randomized controlled trials filter for each database except CENTRAL (Supplemental Tables 1–4). Reference lists of relevant studies and reviews were hand searched to identify articles not captured in the initial search. All databases except CENTRAL were searched again on 27 January 2022 using the same strategy.

### Eligibility criteria

Inclusion criteria included English-language, published, randomized controlled trials that examined the effects of orally ingested probiotics, prebiotics, or synbiotics on the incidence, duration, or severity of GTIs in adults aged 18–65 y. For this review, probiotics were defined as any live population of unicellular microorganisms classified to at least the species level being studied for a health benefit. Prebiotics were defined as any nondigestible saccharide fermented by the gut microbiota, and synbiotics were defined as any combination of probiotics and prebiotics according to the definitions used in this study. Exclusion criteria included the following: abstracts, conference proceedings, clinical trial registrations, and other gray literature; intervention periods of <1 wk total in duration; an unmatched intervention and placebo, defined as no placebo or either product containing added bioactive ingredients not found in the other; animal or in vitro studies; mean or median age of the study population not between 18 and 65 y; studies of hospitalized patients or cohorts with an immunodeficiency, autoimmune, or other immune system disorder; populations taking immune-modulating therapies or antibiotics; and studies examining treatment (e.g., *Helicobacter pylori*, *C. difficile*, and infectious diarrhea) rather than the prevention of GTIs.

During the review, it was determined that few screened studies included medically diagnosed GTIs as an outcome and instead more frequently relied on self-report of GTI-related symptoms (e.g., diarrhea, nausea, and vomiting) that were assessed by a questionnaire. In addition, screened studies commonly administered questionnaires to measure gastrointestinal symptoms, such as bloating, constipation, gas, diarrhea, nausea, and abdominal pain, to assess side effects of an intervention or changes in symptoms of populations with gastrointestinal disorders. Thus, several criteria were established a posteriori regarding the definition of GTIs used for determining study eligibility. These criteria were as follows: studies in which GTIs were diagnosed by a medical professional were eligible for inclusion. When that criterion was not met, studies were eligible for inclusion if GTI or gastrointestinal illness was described as a primary rationale for conducting the study and gastrointestinal symptoms commonly associated with GTI (e.g., diarrhea, fever, and vomiting) were measured. In these cases, the definition of GTIs used was based on self-report of “diarrhea” or similar (e.g., dysentery) because diarrhea was the most consistently and thoroughly reported symptom of studies designed with the intent of measuring GTIs. Studies were excluded if diarrhea could not be dissociated from gastrointestinal symptoms that commonly occur in the absence of infection, such as bloating, flatulence, and abdominal pain. The net effect of these criteria was that studies in which gastrointestinal symptoms were measured primarily as adverse events, studies in which diarrhea was not distinguished from common gastrointestinal symptoms, and studies conducted in populations where gastrointestinal symptoms are generally unrelated to infection (e.g., diarrhea-predominant irritable bowel syndrome and antibiotic-associated diarrhea) were excluded. For the risk-of-bias assessment, studies in which GTIs were diagnosed or diarrhea occurrence was measured daily and defined as ≥3 loose, watery, or unformed stools over 24 h or similar [41] were considered as having a low risk of bias in the measurement of the outcome. If diarrhea was not clearly defined or measured retrospectively, the measurement of the outcome was considered as having a high risk of bias.

### The selection process and data collection

Abstracts identified by each search were uploaded into Covidence systematic review software (Veritas Health Innovation), and duplicate entries were removed using the software’s automated system. The title and abstract of each entry were independently and blindly screened by 2 reviewers (HSF, SDI, LW, ASB, JTA, RTA, DML, AHM, CCW, JPK), and any differences in voting were adjudicated by a third reviewer (JPK or SDI). For any entries that passed this screening, full texts were retrieved and independently screened for eligibility by 2 blinded reviewers (HSF, SDI, LW, ASB, RTA, DML, AHM, CKP, JPK) . Reasons for any exclusions were noted, and any differences were adjudicated by a third reviewer (JPK or SDI). Data extraction and risk-of-bias assessments were completed for each eligible article by 2 blinded reviewers (HSF, SDI, LW, ASB, JTA, JPK). Differences in opinion were discussed, and a third reviewer (JPK) reviewed the data extraction and risk-of-bias assessments to obtain consensus.

The risk-of-bias assessments were completed using version 2 of the Cochrane risk-of-bias tool for randomized trials [42], which assesses risk at the study level within the following domains: bias resulting from the randomization process (domain 1), because of deviations from the intended interventions (domain 2), because of missing outcome data (domain 3), in the measurement of the outcome (domain 4), and in selection of the reported results (domain 5). The risk of bias for each domain was deemed as “low,” “some concerns,” or “high” for domain-specific signaling questions based on the analysis aim of assessing the effect of assignment to the intervention (i.e., ITT effect). Then, results of all 5 domains were used to determine the overall risk of bias [42]. Clinical trial registrations or other publications from the same study were sought to complete assessments as needed.

Data extraction was completed using modified templates created within Covidence. Extracted descriptive information included funding source(s), study author and year of publication, study setting and population, and the number of participants randomized and completing the study. Information extracted for each intervention included intervention type (probiotic, prebiotic, or synbiotic), number of biotics used in the intervention, the strain of probiotic, prebiotic type, treatment dose and duration, form of intervention (capsule, powder, and beverage), and other ingredients used in the intervention or placebo products. Data that allowed for the computation of the effect measures needed for the meta-analysis were also extracted from each study. These data varied by study, were extracted in accordance with how the study authors presented the data, and included the number of subjects with ≥1 GTIs, total number of GTIs, odds/risk/rate ratios of GTI, total days of GTI, days per GTI event, days with GTI per person, and GTI severity. Manual measurement was used for outcome data presented graphically by digitally measuring the locations of means and error bars and converting those values using a height-to-unit ratio determined from digital measurement of the *y*-axis units [43]. Attempts were made to contact corresponding authors when relevant outcomes were not reported or not reported in sufficient detail for inclusion in the meta-analysis.

### Statistical analysis

Based on available data, 3 effect measures were computed and used for statistical analyses: the risk ratio for the number of participants experiencing ≥1 GTIs during the intervention period, the mean and SD for the duration of each GTI episode in individuals who experienced a GTI, and the rate ratio for the total days of illness with a GTI during the intervention period. Risk ratios were derived directly from study reports or, more often, calculated as the proportion of participants with ≥1 GTIs in the intervention group relative to the same proportion in the placebo group. The mean ± SD for the duration of each GTI episode in individuals who experienced a GTI was extracted directly from study reports or obtained from study authors. Rate ratios were calculated as the total days of illness because of GTIs in the intervention group (i.e., total days of GTI illness divided by number of person-years within the intervention period) relative to the same rate in the placebo group. When not provided in the study report, the total days of illness was calculated by multiplying the total number of GTIs in each group by the mean duration of each GTI. Person-years were calculated by multiplying the sample size by intervention duration.

For all 3 effect measures, both intention-to-treat (ITT) and complete case (CC) analyses were conducted. The ITT analysis included all randomly assigned participants. Because attrition resulting in missing outcome data was common across studies (Table 1) [44,59] and most studies reported a CC rather than an ITT analysis, conducting the ITT meta-analysis required that assumptions be made for missing data. The main ITT analysis assumed that no GTI events occurred in participants with missing outcome data. A sensitivity ITT (ITTs) analysis was conducted in which that assumption was modified to be that the risk ratio, rate ratio, or mean ± SD days of illness in participants with missing data were the same as those observed in the CC cohort for the placebo group. The CC analysis included all participants for whom outcome data were reported, regardless of adherence to the intervention.

**TABLE 1:** Characteristics of included studies

Author [reference]; country	Study design; GTI method; incidence ≥1 GTI (%)	Population	*N* randomized (% female); age (y), mean ± SD/ mean (range); attrition (%)	Intervention (daily dose); form of administration	Duration (wk)
**Probiotic**					
*Single strain*					
Hilton et al. [44]; United States	Parallel arm; Qre; NR	International travelers	400 (2); 50 (17–80); 39	*Lactobacillus rhamnosus* GG (2 × 10^9^ CFU/d); capsule	1–3
Oksanen et al. [45]; Finland	Parallel arm; Qre; 44	International travelers	820 (2); 44 ± 14; 14	*L. rhamnosus* GG (2 × 10^9^ CFU/d); powder	1.5
Kollaritsch et al., study 1 [46]; Austria	Parallel arm; Qre; 50	International travelers	319 (47)^1^; 35 ± 17; NR	*Lactobacillus acidophilus* (4 × 10^8^ to 4 × 10^9^ CFU/d); capsule	3, starting day of departure
Kollaritsch et al., study 4 [46]; Austria	Parallel arm; Qre; 36	International travelers	1231 (51)^1^; 42 ± 14; NR	*Saccharomyces cerevisiae* Hansen CBS 5926^2^ (5 × 10^9^ or 10 × 10^9^ CFU/d)^3^; capsule	3, starting 5 d before the travel
Pereg et al. [47]; Israel	Parallel arm; Qre; 14	Military training	541 (0); 18 ± NR; 7	*Lactobacillus casei* DN-114-001 (1 × 10^10^ CFU/d); yogurt	8
Liu et al. [48]; Malaysia	Parallel arm; Qre; 13	Free-living adults during monsoon season	124 (2); (18–60); 11	*Lactobacillus plantarum* DR7 (10^9^ CFU/d); sachet	12
Schröder et al. [56]; Germany	Parallel arm; Qre; 29	Steel workers	242 (0); 42 ± 10; 34	*Lactobacillus reuteri* (5 × 10^8^ CFU/d); chewable tablet	13
Ouwehand et al. [53]; Netherlands	Parallel arm; Med-dx; 100	Healthy adults, oral attenuated ETEC challenge	40 (0); 24 ± 4; 2	*L. acidophilus* ATCC 700396 (2 × 10^9^ CFU/d); capsule	4, starting 2 wk before the ETEC challenge
*Multistrain*					
de dios Pozo-Olano et al., group 1 [51]; United States	Parallel arm; Qre; 0	International travelers	19 (2); NR; 0	*L. acidophilus* and *Lactobacillus bulgaricus* (3.6–7.2 × 10^9^ CFU/d); tablet	1, starting within 48 h before the travel
de dios Pozo-Olano et al., group 2 [51]; United States	Parallel arm; Qre; 29	International travelers	31 (2); NR; 0	*L. acidophilus* and *L. bulgaricus* (3.6–7.2 × 10^9^ CFU/d)^4^; tablet	1, starting within 48 h after arrival
Kalima et al., 90 d [52]; Finland	Parallel arm; Qre and Med-dx; NR	Conscripts attending military training	415 (0); NR; 46	*L. rhamnosus* GG (4x10^10^ CFU/d) and *Bifidobacterium animalis ssp. lactis* BB12 (16 × 10^9^ CFU/d); chewable tablet	13
Kalima et al., 150 d [52]; Finland	Parallel arm; Qre and Med-dx; NR	Conscripts attending military training	568 (0); NR; 76	*L. rhamnosus* GG (4x10^10^ CFU/d) and *B. animalis ssp. lactis* BB12 (16 × 10^9^ CFU/d); chewable tablet	21.5
Haywood et al. [53]; New Zealand	Crossover; Qre; NR	Elite rugby players	30 (0); 25 ± 4; NR	*Lactobacillus gasseri* (2.6 × 10^9^ CFU/d), *Bifidobacterium bifidum* (2 × 10^8^ CFU/d), and *Bifidobacterium longum* (2 × 10^8^ CFU/d); capsule	4
Pumpa et al. [54]; Australia	Parallel arm; Med-dx; 0	Elite rugby players	19 (0); 27 ± 3; 0	Phase A: *L. rhamnosus, L. casei, L. acidophilus, L. plantarum, L. fermentum, Bifidobacterium lactis, B. bifidum,* and *Streptococcus thermophiles* (12 × 10^10^ CFU/d); capsule; phase B: phase A + *Saccharomyces boulardii* (500 mg/d); capsule	6 (phase A); 8 (phase B)
Guillemard et al. [55]; Germany	Parallel arm; Med-dx; 9	Shift workers	1000 (57); 32 ± 9; 4	*L. casei* DN-114-001 (2 × 10^10^ CFU/d), *Streptococcus thermophilus*, and *Lactobacillus delbreuckii* (2 × 10^9^ CFU/d); fermented dairy drink	12
Ten Bruggencate et al. [56]; Netherlands	Parallel arm; Med-dx; 100	Healthy adults, oral attenuated ETEC challenge	60 (0); 25 ± 7; 0	*Lactobacillus helveticus* Rosell-52, *L. rhamnosus* Rosell-11, *Bifidobacterium longum ssp. longum* Rosell-175, and *Saccharomyces cerevisiae var boulardii* CNCM I-1078 (40 × 10^9^ CFU/d); capsule	4, starting 2 wk before the ETEC challenge
**Prebiotic**					
*Fructooligosaccharide*					
Cummings et al. [57]; United Kingdom	Parallel arm; Qre; 42	International travelers	363 (2); 50 ± 13; 33	Fructooligosaccharide (10 g/d); sachet	4, starting 2 wk before the travel
*Galactooligosaccharide*					
Drakoularakou et al. [58]; United Kingdom	Parallel arm; Qre; 31	International travelers	201 (2); 38 ± NR; 21	Galactooligosaccharide (5.5 g/d); sachet	Up to 4, starting 1 wk before the travel^5^
Hasle et al. [59]; Norway	Parallel arm; Qre; 27	International travelers	655 (2); 43 ± NR; 49	Galactooligosaccharide (2.7 g/d); pastille	Up to 3, starting 5 d before the travel^5^
**Synbiotic**					
Virk et al. (14); United States	Parallel arm; Qre; 55	International travelers	251 (2); 49 ± 14; 22	*Enterococcus faecium* SF68 (9 × 10^9^ CFU/d), *Saccharomyces cerevisiae* (10^9^ CFU/d); fructooligosaccaride (dose NR); capsule	4.5 (mean), starting 3 d before the travel^5^

ETEC, enterotoxigenic *Escherichia coli*; GTI, gastrointestinal tract infection; Med-dx, medical diagnosis; NR, not reported; Qre, questionnaire.

1 Number completing study (number randomized not reported).

2 Currently known as *Saccharomyces boulardii* CNCM I-745 [40].

3 Number of events and total sample size in low-dose and high-dose groups combined for meta-analysis and meta-regression. The mean dose used in meta-regression.

4 Midpoint of dose range used for meta-regression.

5 The mean duration used for meta-regression.

Comprehensive Meta-Analysis version 4 (Biostat) was used for all analyses. Random-effects meta-analysis and meta-regression were conducted using the inverse variance method by DerSimonian and Laird [60]. Results are presented as effect measures and associated 95% CIs. Statistical heterogeneity was assessed using the *I*^2^ statistic and was interpreted based on Cochrane handbook recommendations where an *I*^2^ of 30%–50%, 50%–75%, and 75%–100% may suggest moderate, substantial, and considerable heterogeneity, respectively [61]. Publication bias was detected by visually inspecting funnel plots for asymmetry and by Egger test when ≥10 studies were included in the plot [62].

Subgroup meta-analyses and meta-regressions were defined a priori and undertaken to assess potential sources of heterogeneity when the primary analysis suggested moderate heterogeneity or worse. Subgroups considered included the number of probiotic strains used in an intervention (single or multistrain) and study population. Although the original intent of the meta-analyses was to also assess probiotic species-specific and strain-specific effects and specific effects of different prebiotic types, too few studies have used the same probiotic species or strain or prebiotic type to conduct these subgroup analyses. Variables used in meta-regressions included daily dose of probiotic, duration of intervention, and the total dose of probiotic (dose × duration).

Owing to incomplete outcome reporting and a low number of studies for the meta-analysis, vote counting based on the direction of the effect was used as a complementary data synthesis method [63]. A binary metric of benefit or harm was assigned for each outcome based solely on the direction of the effect measure, irrespective of the effect size or *P* value [63]. Then, a binomial probability test was used to test whether the true proportion of effects favoring the intervention was equal to 0.5. Although this approach increased the number of studies included in the data synthesis for several analyses, limitations are that effect sizes and sample sizes are ignored.

## Results

### Study selection and characteristics

**FIGURE 1:**
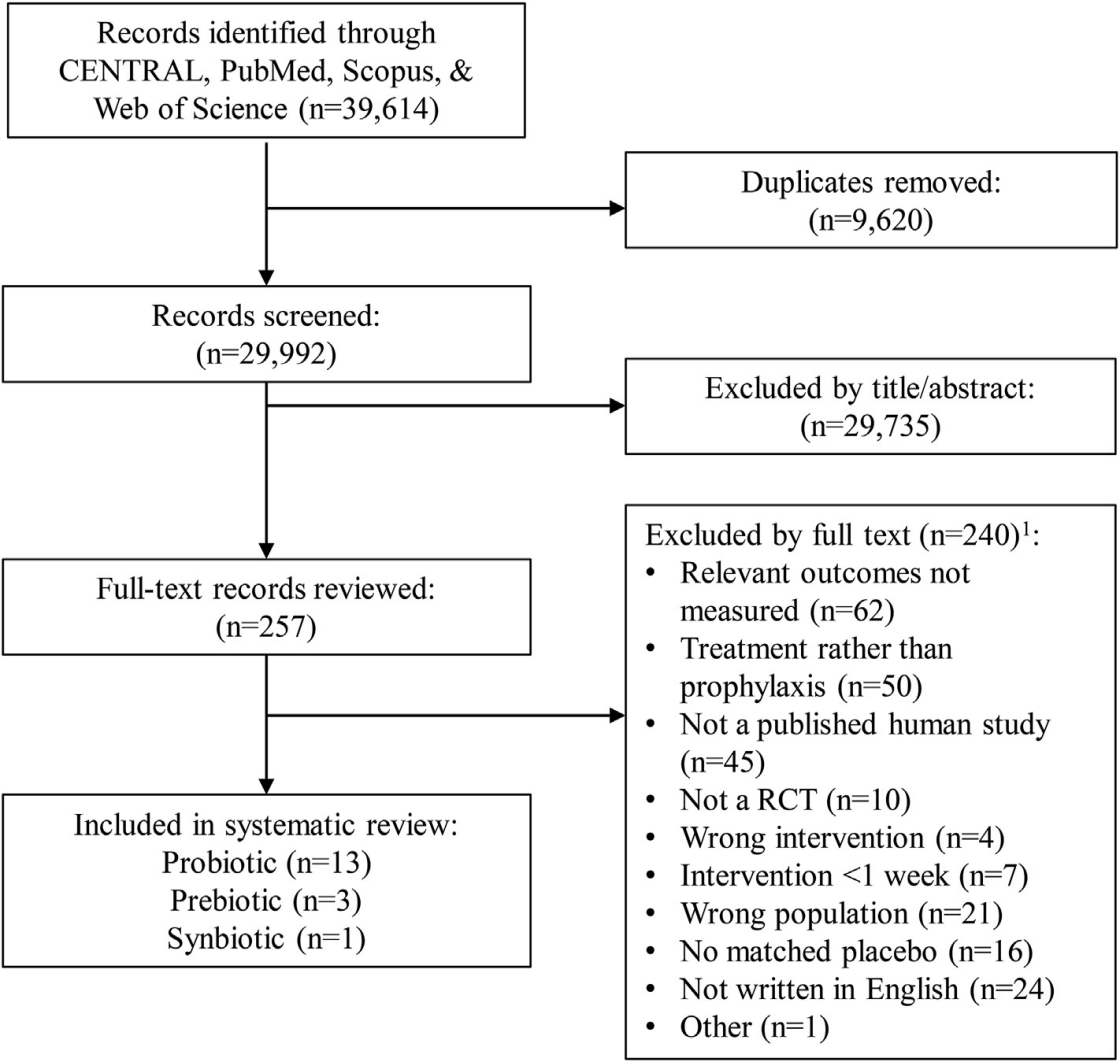
Screening and selection of studies assessing effects of probiotics, prebiotics, or synbiotics on the incidence, duration, or severity of gastrointestinal tract infections (GTIs) in nonelderly adults. RCT, randomized controlled trial. ^1^For several studies, multiple exclusion criteria were applicable, but only 1 criterion was recorded.

A total of 29,992 unique records were identified and screened (Figure 1). Of the 257 full texts reviewed, 240 were excluded based on ≥1 criteria, leaving 17 separate publications describing 20 different studies that were determined to be eligible for inclusion (Table 1). Publication dates of included studies ranged from 1978 to 2020, and studies were conducted in North America (*n* = 4), Europe (*n* = 12), Oceania (*n* = 2), and Asia (*n* = 2). Of the eligible publications, 4 could not be included in meta-analyses owing to data being reported in an unusable format [50,56], insufficient data [53], or an absence of GTI events in the study population [54].

All but one [53] of the included studies followed a parallel-arm design (Table 1). Two studies used a controlled human infection model where subjects were infected with a live but attenuated dose of an enteric pathogen [50,56]. Other studies were conducted with adults living in (*n* = 1) or traveling to (*n* = 10) regions where the risk of GTI was high, military trainees (*n* = 3), athletes in training (*n* = 2), or shift workers (*n* = 2) (Table 1). Only 6 studies reported medical diagnosis of GTI as an outcome [[50], [52], [54], [55], [56]], whereas all other studies relied on self-report and questionnaires. The reported incidence of 1 or more GTIs ranged from 0% [51,54] to 55% [14] and was the highest in studies of travelers (Supplemental Figure 1A). Attrition ranged from 0% [51,54,56] to 76% [52] and was not reported in 2 studies [46,53] (Table 1).

Included studies tested 13 different orally ingested probiotic formulations, 2 different orally ingested prebiotic types, and 1 orally ingested synbiotic formulation (Table 1 and Supplemental Table 5). Probiotic interventions included both single-strain (*n* = 7) [[44], [45], [46], [47], [48], [49], [50]] and multistrain (*n* = 6) [[51], [52], [53], [54], [55], [56]] products. All interventions containing probiotics but 2 [14,46) included at least 1 *Lactobacillus* strain (Table 1 and Supplemental Table 5). Many studies did not report strain identification, and among those that did, few tested the same strains within single-strain or multistrain interventions in >1 study. Exceptions included *Lactobacillus rhamnosus GG* (*n* = 4 studies) and *Lactobacillus casei* DN-114-001 (*n* = 2 studies). Several species of probiotic were tested as single-strain interventions or within multistrain interventions in multiple studies, including *Lactobacillus acidophilus* (*n* = 4 studies), *Lactobacillus bulgaricus* (*n* = 2 studies), *L. casei* (*n* = 3 studies), *Lactobacillus plantarum* (*n* = 2 studies), *L. rhamnosus* (*n* = 6 studies), *Bifidobacterium animalis ssp. lactis* (*n* = 2 studies), *Bifidobacterium bifidum* (*n* = 2 studies), *Bifidobacterium longum* (*n* = 2 studies), and *Saccharomyces cerevisiae* (*n* = 4 studies). Prebiotics tested included FOSs (*n* = 2 studies) and GOSs (*n* = 2 studies) (Table 1 and Supplemental Table 5). Doses of probiotic interventions ranged from 5 × 10^8^ to 12 × 10^10^ CFUs/d, and prebiotic interventions ranged from 2.7 to 10 g/d (Table 1). Intervention durations ranged from 8 to 150 d (Table 1), and several studies started interventions from 2 to 14 d before initiating infection with an enteric pathogen [50,56] or exposure to an environment where risk of exposure to enteric pathogens was considered high [14,[44], [45], [46],^51^,[57], [58], [59]]. Within probiotic studies, median intervention doses were approximately 20 times higher (4.0 × 10^10^ CFU/d compared with 2.1 × 10^9^ CFU/d) (Supplemental Figure 1B) and median study durations were twice as long (8 wk compared with 4 wk) (Supplemental Figure 1C) in studies using multistrain versus single-strain interventions.

### Effects of orally ingested probiotics on GTI incidence, duration, and severity

**FIGURE 2:**
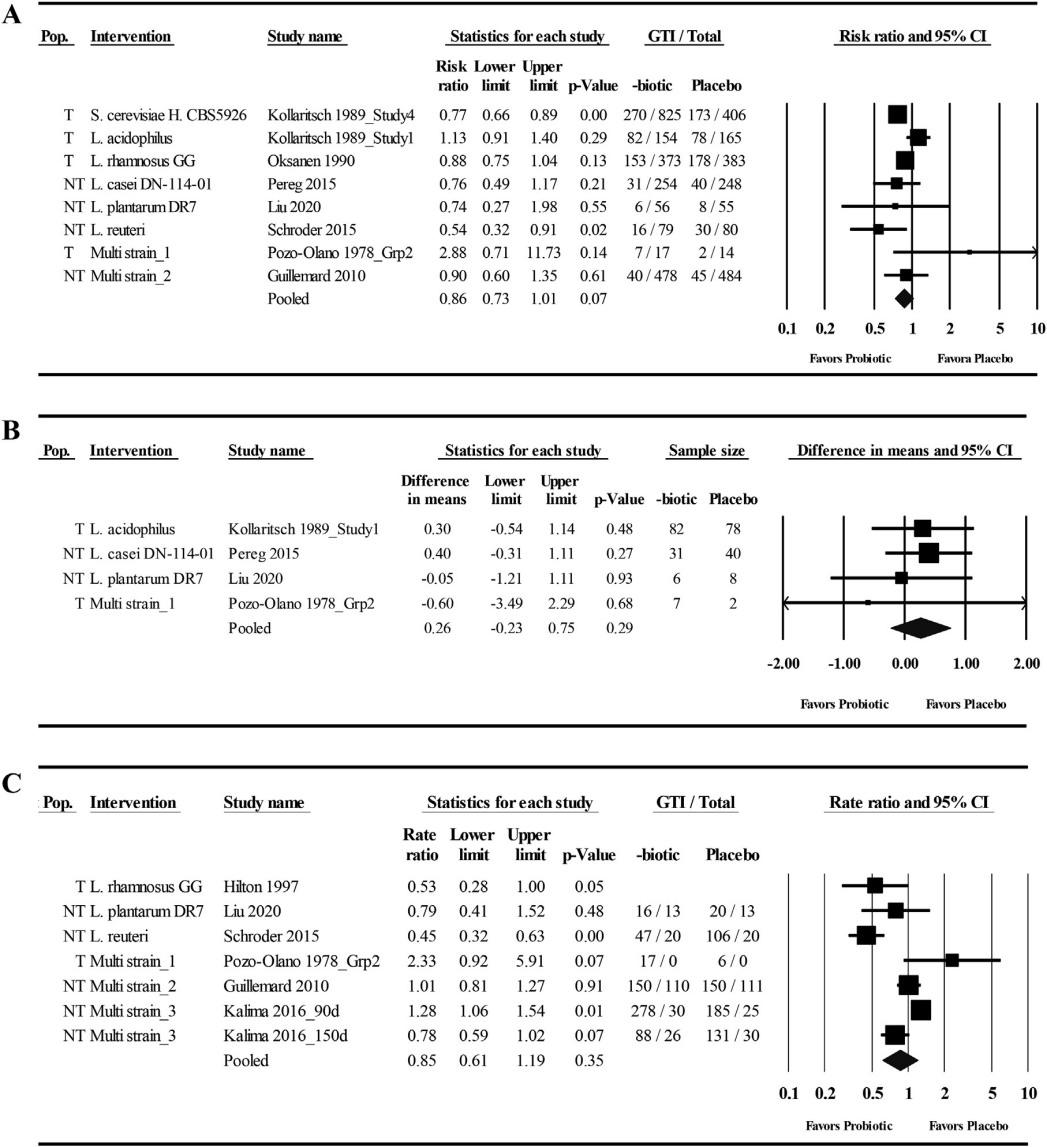
Forest plots of the effects of orally ingested probiotics compared with placebo on the incidence and duration of gastrointestinal tract infections (GTIs) in nonelderly adults. (A) Risk of experiencing ≥1 GTIs (*I*^2^ = 51.8, *P* = 0.04); (B) days per individual GTI episode (*I*^2^ = 0, *P* = 0.86); (C) total days of illness with GTI (*I*^2^ = 84.1, *P* < 0.001). (A–C) Complete case random-effects meta-analysis using the inverse variance method by DerSimonian and Laird [60]. Individual study effect estimates (squares; sized by study weight) and pooled effects (diamonds) are plotted. Lower and upper limits are 95% CIs. Grp, group; H., Hansen; L., *Lactobacillus*; NT, nontraveler; Pop., population; S., *Saccharomyces*; T, travelers. Multistrain_1: *L. acidophilus, L. bulgaricus*; Multistrain_2: *L. casei* DN-114-001, *Streptococcus thermophilus, L. delbreuckii;* Multistrain_3: *L. rhamnosus* GG, *B. animalis ssp. lactis* BB12.

Six studies that examined the effects of orally ingested probiotics compared with those of placebo on GTI incidence provided sufficient data for both of the ITT analyses and the CC meta-analysis [45,[47], [48], [49],^51^,^55^]. Two additional studies of travelers that used single-strain interventions and which were reported in the same manuscript [46] provided data for only the CC analysis. Across the 3 analyses, mean effect sizes favored probiotics and ranged from a 14% to 17% risk reduction, with 95% CIs spanning a 32% risk reduction to a 2% increase in risk (Table 2, Figure 2A**,** and Supplemental Figure 2A,B). Substantial heterogeneity was observed in the CC analysis and was more than double the heterogeneity observed in the ITT analyses (Table 2). Removing the 2 studies that were included in the CC analysis but not the ITT analyses resulted in lower heterogeneity (*I*^2^ = 22.6; *P* = 0.26). A subgroup analysis using the study population as the grouping factor (travelers or other) revealed low heterogeneity in the CC meta-analysis of studies conducted in nontraveler populations but considerable heterogeneity in the traveler cohorts (Table 2). A subgroup analysis of single-strain interventions did not lower heterogeneity, and neither the dose nor the duration of interventions were associated with GTI risk in CC analyses (Table 2). Three additional studies reported data relevant to GTI incidence as defined in this study but could not be included in the meta-analyses. Of those studies, all used multistrain interventions, 2 reported no GTI events in small cohorts of competitive athletes [54] and travelers [51], and 1 used a crossover design and reported that fewer competitive athletes experienced ≥1 GTI when receiving the probiotic (4/30) relative to the placebo (5/30) [53]. Collectively, of the 9 studies that provided data on GTI incidence and recorded at least 1 event, 7 favored the probiotic interventions (binomial probability test, *P* = 0.18). The 2 studies that did not were conducted with cohorts of travelers [46,51].

**TABLE 2:** Meta-analyses and meta-regression of the effects of orally ingested probiotics compared with placebo on the risk of experiencing ≥1 gastrointestinal tract infections in nonelderly adults

	Studies (*n*)	Risk ratio (95% CI)	*P*	*I*^2^	*P*
**Overall effect**^1^					
*ITT analysis*^2^	6	0.83 (0.68, 1.02)	0.07	22.0	0.27
*ITTsanalysis*^2^	6	0.86 (0.76, 0.98)	0.02	0	0.47
*CC analysis*	8	0.86 (0.73, 1.01)	0.07	51.8	0.04
**Subgroup**^1^					
*CC analysis*					
Number of strains					
Single	6	0.84 (0.71, 1.00)	0.05	56.9	0.04
Population					
Travelers	4	0.93 (0.75, 1.16)	0.51	72.3	0.01
Nontravelers	4	0.75 (0.58, 0.96)	0.02	0	0.51
**Meta-regression**^3^	**Studies (*****n*****)**	***β*****(95% CI)**	***P***	***I***^2^	***P***^4^
*CC analysis*					
Dose (log_10_ CFU/d)	8	0.003 (−0.22, 0.23)	0.98	57.5	0.03
Duration (d)	8	−0.004 (−0.01, 0.002)	0.18	52.4	0.05
Total dose (log_10_ CFU/d)	8	−0.05 (−0.25, 0.16)	0.67	56.3	0.03

CC, complete case; ITT, intention-to-treat assuming no cases among participants with missing data; ITTs, intention-to-treat assuming the risk ratio among participants with missing data matches the risk ratio of the control group from the CC analysis.

1 Random-effects meta-analysis using the inverse variance method by DerSimonian and Laird.

2 Does not include study 1 or study 4 of Kollaritsch et al. [46] owing to missing information on the total number of participants enrolled.

3 Random-effects meta-regression using the inverse variance method by DerSimonian and Laird [60] with log risk ratio as the dependent variable.

4 *I*^2^ calculated from the goodness-of-fit test.

Four studies that examined the effects of orally ingested probiotics compared with placebo on the duration of individual GTI episodes provided data for the meta-analysis [[46], [47], [48],^51^]. One of the studies could not be included in the ITTs analysis owing to not disclosing the number of individuals randomized [46]. The mean effect sizes indicated a 0.15– 0.26 d/event increase in episode duration with probiotic interventions, with 95% CIs spanning a 0.29 d/event shorter duration to a 0.75 d/event longer duration and low heterogeneity between studies (Table 3, Figure 2B**,** and Supplemental Figure 3A,B). Two additional studies of military trainees that did not provide sufficient data for the meta-analysis reported that the mean episode duration was either reduced by 0.4 d or increased by 0.5 d in cohorts receiving probiotics relative to placebo (variability not reported) [52]. Collectively, of the 6 studies identified, 3 reported effects favoring the probiotic interventions.

**TABLE 3:** Meta-analyses of the effects of probiotics compared with those of placebo on the duration of gastrointestinal tract infections (d/episode) in nonelderly adults

	Studies (n)	Mean difference (d/episode) (95% CI)	*P*	*I*^2^	*P*
*ITT and CC analysis*	4	0.26 (−0.22, 0.75)	0.29	0	0.86
*ITTsanalysis*^1^	3	0.15 (−0.29, 0.59)	0.50	0	0.77

CC, complete case; ITT, intention-to-treat assuming no cases among participants with missing data; ITTs, intention-to-treat assuming the risk ratio among participants with missing data matches the risk ratio of the control group from the CC analysis.

Random-effects meta-analysis using the inverse variance method by DerSimonian and Laird.

1 Does not include study 1 of Kollaritsch et al. [46] owing to missing information on the total number of participants enrolled.

Six studies that examined the effects of orally ingested probiotics compared with placebo on the total days of GTI illness, which reflects both the total number of GTI events and duration of each event, provided data for the ITT meta-analyses [46,48,49,51,52,55]. One additional study of travelers that used a single-strain intervention [46] provided data for only the CC analysis. Across the 3 analyses, mean effect sizes ranged from a 4% to 15% reduction in the rate of illness, with 95% CIs spanning a 40% reduction to a 43% increase in the rate (Table 4, Figure 2C**,** and Supplemental Figure 4A,B). Considerable heterogeneity was observed in all analyses (Table 4). Subgroup analyses revealed that heterogeneity remained substantial or considerable in studies of mutistrain interventions and nontraveler populations but was low in studies of single-strain interventions (Table 4). The mean effect size favored probiotics in studies of single-strain interventions, although only 3 studies were included. Daily supplementation dose was positively correlated with the log rate ratio for total days of illness in the CC and ITTs meta-regressions (Table 4). Of the 7 studies included in the meta-analyses, 4 reported effects favoring probiotics (binomial probability test, *P* = 0.37), and the effects reported in all 3 studies of single-strain interventions favored probiotics (binomial probability test, *P* = 0.25).

**TABLE 4:** Meta-analyses and meta-regressions of the effects of orally ingested probiotics compared with placebo on the rate of total days of illness from gastrointestinal tract infections in nonelderly adults.

	Studies (*n*)	Rate ratio (95%CI)	*P*	*I*^2^	*P*
**Overall effect**^1^					
*ITT analysis*^2^	6	0.93 (0.60, 1.43)	0.73	90.9	<0.001
*ITTsanalysis*^2^	6	0.96 (0.78, 1.18)	0.69	76.7	0.001
*CC analysis*	7	0.85 (0.61, 1.19)	0.35	84.1	<0.001
**Subgroup**^1^					
No. of strains					
*ITT analysis:* multiple	4	1.14 (0.73, 1.78)	0.56	89.6	<0.001
*ITTsanalysis:* multiple	4	1.06 (0.91, 1.25)	0.45	60.4	0.06
*CC analysis:* multiple	4	1.08 (0.81, 1.44)	0.59	74.6	0.01
Single	3	0.52 (0.38, 0.70)	<0.001	10.7	0.33
Population: Nontravelers					
*ITT analysis*	5	0.83 (0.53, 1.31)	0.42	92.3	<0.001
*ITTsanalysis*	5	0.93 (0.76, 1.13)	0.44	78.0	0.001
*CC analysis*	5	0.83 (0.58, 1.18)	0.30	87.1	<0.001
**Meta-regression**^3^	**Studies (*****n*****)**	***β*****(95% CI)**	***P***	***I***^2^	***P***^4^
*ITT analysis*					
Dose (log_10_ CFU/d)	6	0.26 (−0.20, 0.72)	0.27	89.6	<0.001
Duration (d)	6	−0.01 (−0.02, 0.003)	0.16	90.6	<0.001
Total dose (log_10_ CFU/d)	6	0.07 (−0.35, 0.49)	0.74	91.0	<0.001
*ITTsanalysis*					
Dose (log_10_ CFU/d)	6	0.18 (0.002, 0.36)	0.05	63.3	0.03
Duration (d)	6	−0.04 (−0.01, 0.004)	0.33	80.2	0.001
Total dose (log_10_ CFU/d)	6	0.12 (−0.07, 0.30)	0.23	71.8	0.01
*CC analysis*					
Dose (log_10_ CFU/d)	7	0.30 (0.01, 0.59)	0.04	74.0	0.002
Duration (d)^2^	6	−0.01 (−0.02, 0.003)	0.19	86.4	<0.001
Total dose (log_10_ CFU/d)^2^	6	0.12 (−0.20, 0.44)	0.47	83.3	<0.001

CC, complete case; ITT, intention-to-treat assuming no cases among participants with missing data; ITTs, intention-to-treat assuming the risk ratio among participants with missing data matches the risk ratio of the control group from the CC analysis.

1 Random-effects meta-analysis using the inverse variance method by DerSimonian and Laird [60].

2 Does not include Hilton et al. [44] owing to incomplete information on the total number of participants enrolled and study duration.

3 Random-effects meta-regression using the inverse variance method by DerSimonian and Laird [60] with log rate ratio as the dependent variable.

4 *I*^2^ calculated from the goodness-of-fit test.

Few studies have reported data on GTI symptom severity and those that did frequently used multiple metrics of severity without calculating a composite global severity score. Therefore, a meta-analysis was not conducted. Among the 4 studies that provided data on illness severity, 1 reported an effect that favored probiotics [55] and 3 reported effects that favored probiotics on some indices but not others [46,47,55].

### Effects of orally ingested prebiotics on GTI incidence, duration, and severity

**FIGURE 3:**
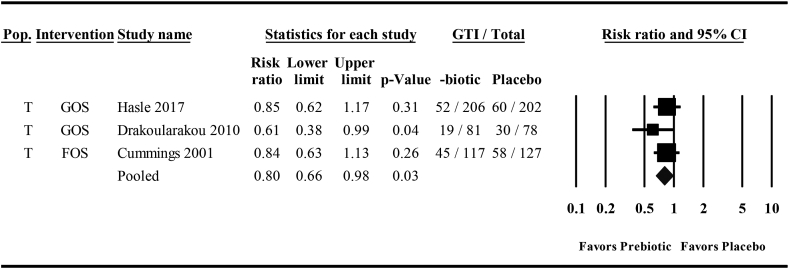
A forest plot of the effects of orally ingested prebiotics compared with placebo on the risk of experiencing ≥1 gastrointestinal tract infections (GTIs) in nonelderly adults. A complete case random-effects meta-analysis using the inverse variance method by DerSimonian and Laird [60]. Individual study effect estimates (squares; sized by study weight) and pooled effects (diamond) are plotted. *I*^2^ = 0, *P* = 0.48. Lower and upper limits are 95% CIs. FOS, fructooligosaccharide; GOS, galactooligosaccharide; Pop., population; T, traveler.

Three studies examined the effects of orally ingested prebiotics compared with placebo on GTI incidence and were included in the meta-analysis [45,47,48]. All studies assessed GTI in travelers, and 2 used the same GOS intervention [58,59], whereas the other used FOS [57]. Across the 3 meta-analyses, mean effect sizes ranged from a 13% to 21% risk reduction with 95% CIs spanning a 37% risk reduction to a 1% increase in risk with a low heterogeneity between studies (Table 5, Figure 3, and Supplemental Figure 5A,B).

**TABLE 5:** Meta-analyses of the effects of orally ingested prebiotics compared with placebo on the risk of experiencing 1 or more gastrointestinal tract infections in nonelderly adults.

	Studies (*n*)	Risk ratio (95% CI)	*P*	*I*^2^	*P*
*ITT analysis*	3	0.79 (0.63, 0.97)	0.03	0	0.61
*ITTsanalysis*	3	0.87 (0.74, 1.01)	0.07	0	0.47
*CC analysis*	3	0.80 (0.66, 0.98)	0.03	0	0.48

CC, complete case; ITT, intention-to-treat assuming no cases among participants missing data; ITTs, intention-to-treat assuming the risk ratio among participants missing data matches the risk ratio of the control group from the CC analysis. Random-effects meta-analysis was performed using the inverse variance method by DerSimonian and Laird [60].

Reporting of GTI duration and severity varied across the prebiotic studies, which precluded meta-analysis of these outcomes. Effects on the duration of diarrheal episodes favored the prebiotic (mean difference [95% CI] = 2.2 d/event [0.7, 3.7], ITT and CC analyses; 1.1 d/event [−0.1, 2.3], ITTs analysis) in one of the GOS studies [58] but favored the placebo by 0.9 d/event (variability not reported) in the other [59], and episode duration was not reported in the FOS study [57]. Effects on illness severity favored the prebiotic across multiple indices in 1 GOS study [59], favored the prebiotic for some but not all symptoms in the other GOS study [58], and could not be distinguished from side effects in the FOS study [57].

### Effects of orally ingested synbiotics on GTI incidence, duration, and severity

Only 1 study that examined the effects of an orally ingested synbiotic compared with placebo on GTI incidence, duration, or severity was identified [14]. In that study of healthy adult travelers, the effects on the incidence of TD favored the placebo (CC risk ratio: 1.03; 95% CI: 0.79, 1.32) [14]. Effects on event duration and illness severity were not reported.

### Effects of supplementation timing on GTI incidence

Several studies included in this review raised the prospect that the initiation of dosing relative to the timing of travel to high-GTI–risk regions may affect product effectiveness [46,51,59]. To assess this possibility, a posteriori analysis of probiotic, prebiotic, and synbiotic studies conducted in international traveler cohorts [14,45,46,51,[57], [58], [59]] was conducted. Using the CC data to maximize the sample size, no association between the number of days the intervention was administered before travel and the log of the risk ratio for experiencing ≥1 GTIs was observed (*β* = −0.02; 95% CI: −0.05, 0.01; *P* = 0.14; *I*^2^ = 43.9; *P* = 0.10).

### Risk of bias and publication bias

**FIGURE 4:**
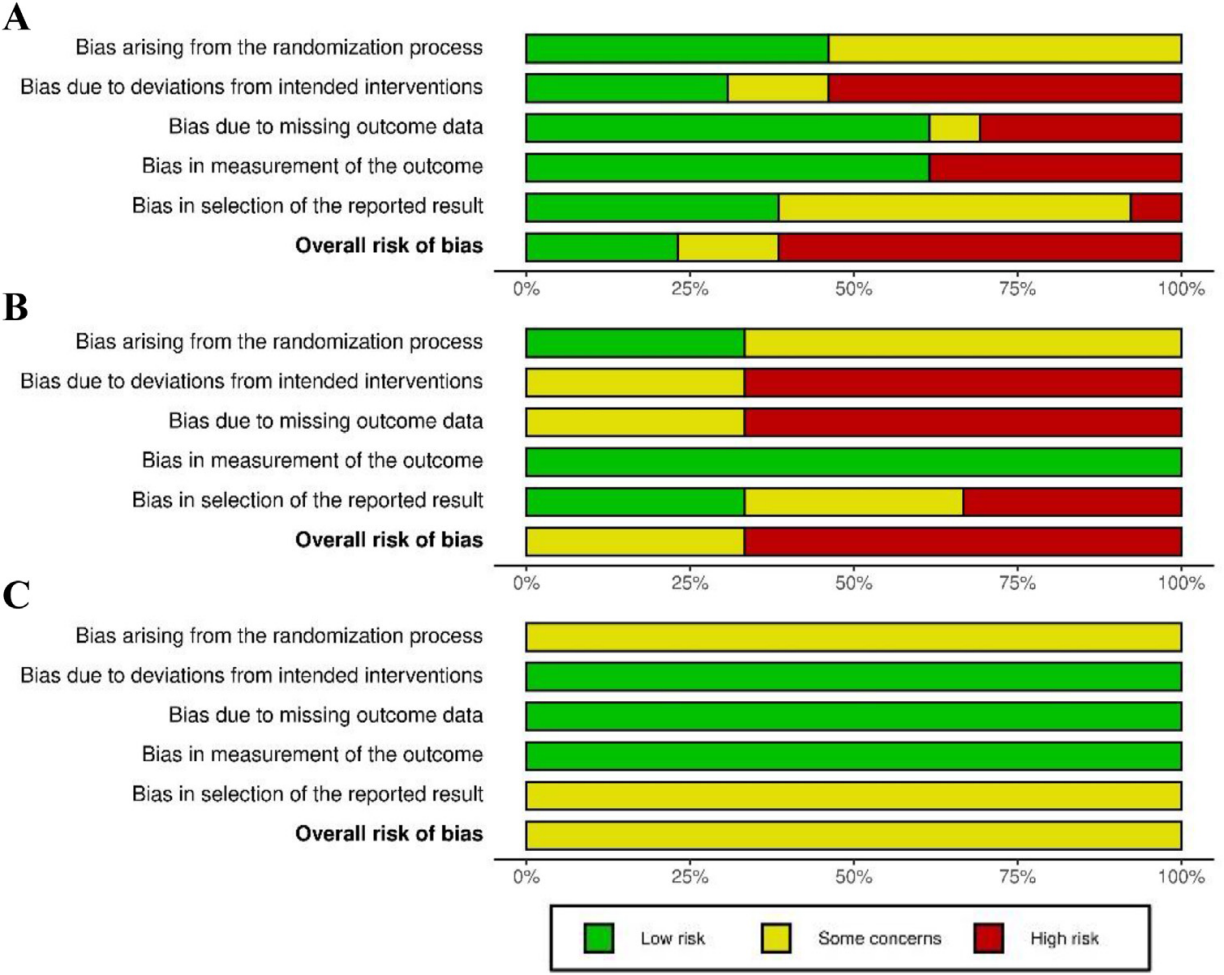
Risk-of-bias assessment for all studies included in the systematic review of the effects of probiotics (A), prebiotics (B), and synbiotics (C) on the incidence, duration, and severity of gastrointestinal tract infections (GTIs) in nonelderly adults, assessed using the Cochrane risk-of-bias assessment tool version 2.0. Plot produced using robvis [64].

The overall risk of bias was determined to be high for most studies included in the analysis and was often attributable to deviations from the intended intervention resulting from high attrition, missing outcome data, and bias in methods used to measure GTIs (Figure 4 and Supplemental Figure 6) [64]. To assess publication bias, funnel plots were generated using results from the CC analyses and by combining all available studies for each outcome regardless of the intervention type (Supplemental Figure 7). Visual inspection of the funnel plots did not provide any clear evidence of publication bias. Moreover, Egger test did not provide evidence of publication bias when applied to the outcome of GTI incidence (*P* = 0.49). Tests for funnel plot asymmetry were not conducted for episode duration and total duration of illness owing to the low number of studies in each analysis.

## Discussion

Several meta-analyses have concluded that orally ingested probiotics or synbiotics show promise as treatment options for GTIs [23,27,29,30,32,33]; however, far fewer meta-analyses have examined the prophylactic use of orally ingested probiotics, prebiotics, or synbiotics for reducing the GTI burden in adult populations. In this systematic review, 17 publications reporting results of 20 randomized controlled trials that assessed the prophylactic effects of these gut microbiota-targeted interventions on GTI incidence, duration, or severity among nonelderly adults were identified. Meta-analyses indicated that studies of both probiotic and prebiotic interventions showed modest benefit for reducing the risk of experiencing ≥1 GTIs. The effects of the interventions on GTI duration, total days of illness, and GTI severity were generally null or unable to be meta-analyzed owing to a lack of available studies. The study population and number of probiotic strains included in an intervention were identified as sources of heterogeneity for some outcomes. However, the number of studies available for meta-analysis, and especially for meta-analysis of prebiotics, synbiotics, and subgroups, was low, heterogeneity was substantial or worse for many analyses, CIs for effect estimates all approached or included an estimate of no effect, the risk of bias for most studies was high, and the effects of specific probiotic strains and prebiotic types could not be assessed. Taken together, these findings suggest that the prophylactic use of certain probiotic and prebiotic interventions has potential for reducing GTI incidence in some adult populations but highlight a need for more high-quality studies.

Meta-analyses suggested that the prophylactic use of oral probiotics reduced the risk of experiencing ≥1 GTIs by ∼15%. However, uncertainty around that estimate indicated that the true effect could range from what would be considered an appreciable risk reduction of ∼25%–30% [61] to no effect. Subgroup analyses indicated that heterogeneity in those estimates was largely attributable to the study population. The 4 studies conducted in international travelers at risk for TD reported both decreases and increases in risk, whereas the 4 conducted in other populations consistently reported risk reductions. In comparison, other meta-analyses have reported that prophylactic use of orally ingested probiotics resulted in a 8% (risk ratio: 0.92; 95% CI: 0.79, 1.05), 6 studies [35]) to 15% (risk ratio: 0.85; 95% CI: 0.79, 0.92, 7 studies [39]) reduction in the risk of TD in adults. Discrepancies in effect sizes across the 3 analyses are attributable to differences in inclusion criteria and data extraction and analytic methods, although CIs do overlap. In other meta-analyses, prophylactic probiotic use has been found to reduce the risk of diarrhea of unknown etiology by 34% [8] and reduce the risk or odds of *C. difficile*–associated diarrhea and/or *C. difficile* infection by 60%–70% among adults and children [[35], [36], [37]]. That the mean effect sizes are much larger than those observed in this study and in TD studies may be attributable to the inclusion of children and other populations at high risk of infection, the ability to select probiotics targeted toward a specific pathogen, or greater efficacy of probiotics in patients with less-developed commensal microbiota or who have been exposed to gut microbiota-depleting antibiotics. In addition, in several previous meta-analyses, heterogeneity in overall effects and effect sizes has been associated with different probiotic strains [8,[35], [36], [37]] and causes of infection [8,35]. For example, McFarland et al. [39] reported a reduction in TD risk in 2 studies using *S. cerevisiae* Hansen CBS 5926 and in 2 studies using *L. rhamnosus* GG but an increase in risk in 3 studies using *L. acidophilus*. Taken together, these meta-analyses suggest that oral prophylaxis with probiotics may reduce the risk of GTI in adults, and particularly the risk of GTI-associated diarrhea. However, individual species or strains may vary in effectiveness based on the nature and source of risk. This is consistent with the concept that the effects of probiotics are often strain and disease (or pathogen) specific [65]. Unfortunately, the role of species-specific and strain-specific effects on GTI incidence could not be addressed in this analysis owing to incomplete characterization of strain identity in multiple studies and a lack of multiple studies using the same probiotic species or strain.

Oral probiotics were not found to reduce the duration of individual GTI episodes or the overall rate of total days of illness because of GTI. By contrast, previous meta-analyses have shown that probiotics reduce the duration of acute diarrhea when used as a treatment strategy in infants, children, and hospitalized patients [[21], [22], [23],^26^,^27^]. Results of this analyses should be interpreted cautiously given that only 4 studies reported on GTI episode duration and the considerable heterogeneity observed in meta-analyses of the illness rate. In analyses exploring the latter observation, heterogeneity remained considerable in studies conducted within nontraveler cohorts, whereas the 3 studies conducted with single-strain interventions consistently reported reductions in illness rates with low heterogeneity across studies. That the studies using single-strain interventions also provided lower daily probiotic doses may explain the positive association between the daily dose and the rate of illness in the CC and ITTs meta-regressions. These observations are noticeably opposite of the effect of the same factors on heterogeneity in the analysis of GTI incidence. Whether that contrast indicates that the effects of probiotic strain count and study population differ based on outcome or reflect the fact that different studies using different interventions were included in each analysis is unclear. However, half or more of the 4 studies using multistrain interventions reported the effects of GTI risk or rate that favored the placebo, whereas 6 of 7 studies conducted with single-strain interventions reported effects favoring the probiotics (Figures 2A, C). These observations should not be construed as indicating that single-strain interventions are superior to multistrain interventions. Nonetheless, they do raise the possibility that multistrain interventions might have more heterogeneous effects than single-strain interventions. Possible reasons could be resource competition among multiple strains or antagonistic effects that reduce the efficacy of any one strain. Single -strain interventions may also be selected to target particular conditions or gastrointestinal pathogens, whereas multistrain interventions may be more often studied with the intent of general immune enhancement so as to reduce the risk of any common infection. Regardless, more research is clearly needed to identify which probiotic strains and strain combinations are most effective against specific pathogens. Importantly, as has been suggested elsewhere [66,67], these studies should not assume that more strains or higher doses beyond a minimal threshold equate to greater effectiveness.

Only 3 studies evaluating the effectiveness of 2 separate oral prebiotics for GTI prevention were identified, and all were conducted in traveler cohorts. In those studies, prebiotics consistently reduced the risk of experiencing one or more episodes of TD, with an estimated mean risk reduction of ∼15%–20%, though uncertainty around those estimates indicated that the true effect could range from a ∼25%–35% risk reduction to no effect. These findings show greater precision around a similar estimate of the mean effect when compared to McFarland et al. [39] who reported wide uncertainty in the effects of prebiotics on TD risk among adult travelers (risk ratio = 0.83 [95% CI: 0.58, 1.18], 3 studies). Importantly, our study population was slightly different as we excluded a study by Krokowicz et al. [68] that tested sodium butyrate, which is not a recognized prebiotic. We also included one study published after McFarland et al.’s [39] analysis, which was particularly notable in that higher adherence to the GOS intervention was shown to be associated with a greater reduction in the odds of TD [59]. The present findings, when considered in the context of the few studies available for inclusion and concerns regarding risk of bias among those studies, indicate that future studies should continue to investigate the potential role of oral prebiotics in GTI prevention and assess whether the observed effects are comparable across other populations, exposures, and types of prebiotics. From a practical standpoint, prebiotic interventions could provide important alternatives to certain probiotics given the similar effect sizes observed here and because prebiotics may offer logistical advantages to some probiotic products, such as greater stability, longer shelf-life, and not requiring refrigeration. Future studies should also consider an empirically-based approached to combining probiotics and prebiotics (i.e., synbiotics) given that both intervention types separately demonstrated favorable effects on GTI incidence in the present analysis, that mechanisms and sites of action may differ, recent evidence that synbiotics are more effective than probiotics in reducing the duration of diarrhea when used to treat acute gastroenteritis [22], and the notable lack of studies investigating synbiotics for the prevention of GTI in nonelderly adults. If multiple probiotic strains are to be included, care should be taken to consider how interactions among the strains could increase or decrease product efficacy [67].

### Strengths and limitations

The strengths of this systematic review and meta-analysis include a comprehensive search strategy, investigation into sources of heterogeneity, and inclusion of both ITT and CC analyses. However, the results must be interpreted within the context of several limitations. The foremost is the low number of studies available for inclusion. That limitation reduced the power for each analysis, and, as a result, mean effect estimates need to be interpreted cautiously and within the context of uncertainty around the mean effect size. In particular, the results of subgroup analyses, meta-regressions, and meta-analyses of prebiotic interventions should be considered hypothesis generating rather than definitive because of the low power for those analyses. Relatedly, the same probiotic and prebiotic interventions were not tested in more than one study with the exception of *L. rhamnosus* GG (2 studies) and GOS (2 studies). That limitation precluded addressing an a priori aim of investigating strain-specific effects of probiotics and compound-specific effects of prebiotics on the GTI burden, which may be sources of unexplained heterogeneity in several of the analyses. Previous meta-analyses and systematic reviews have reported strain-specific efficacy of probiotic interventions against particular illnesses [65,67,69,70]. These strain-specific effects are likely mediated, in part, by unique mechanisms of action specific to individual probiotic strains [15]. For these reasons, some have argued that meta-analyzing probiotic interventions any less precisely than at the strain level should be avoided because doing so could lead to an overgeneralization or underestimation of effectiveness [65].The same concept may be applicable to prebiotics and candidate prebiotics because certain chemical structures will be metabolizable by only a subset of bacteria [71]. On the other hand, the existence of shared mechanisms of action of probiotics and prebiotics for promoting health and reducing the illness burden is also generally acknowledged. For probiotics, these “core benefits” may include reduced luminal pH, colonization resistance, and competitive exclusion of pathogens [15]. For prebiotics, core benefits include propagating health-promoting commensal bacterial strains and reducing luminal pH by providing a substrate for SCFA production [19]. These core benefits could all impact GTI risk independent of strain- or compound-specific effects. Therefore, meta-analyses that synthesize data from probiotic or prebiotic intervention studies using different probiotic strains or prebiotic types can provide valuable insight regarding the potential effectiveness of these interventions for reducing the GTI burden, especially when few studies are available. However, the evidence base regarding -biotic interventions for reducing the GTI burden would clearly be strengthened by the addition of well-designed clinical trials that aim to reproduce the beneficial effects of specific probiotic strains or mixtures, prebiotic types, and their combinations.

Additional limitations pertain to the design and bias present within the studies included in the analysis. First, few studies included diagnosed infections as an outcome, which required that results from symptom diaries be used to identify GTIs. That approach may have resulted in misclassification bias given that diarrhea and other gastrointestinal symptoms can result from noninfectious causes. Second, the lack of consistently defined, extractable data on GTI severity precluded a cohesive synthesis and meta-analysis of illness severity. Third, the risk of bias was high for many of the included studies, most often because of high attrition, resulting in nonadherence to the intervention and missing outcome data. Few studies used an appropriate ITT analysis to address the issue, instead reporting a CC analysis without describing adherence to the intervention. The present analysis made 2 assumptions regarding missing data, that either no GTI events occurred in participants with missing data (ITT analysis) or that the risk, mean duration, or rate of GTIs were the same as in the CC data from the placebo group (ITTs analysis). Both assume no difference between the intervention and placebo groups, which would bias results toward the null.

## Conclusions

Few studies have assessed the prophylactic effects of orally ingested probiotics, or prebiotics and synbiotics, in particular, on GTI incidence, duration, or severity in nonelderly adults. The limited available evidence suggests that orally ingested probiotics and prebiotics may reduce the risk of GTI in nonelderly adults, while effects on illness duration and severity appear null or are unclear because of a lack of studies. A high risk of bias was observed for most studies, and imprecision in effect estimates indicated that the magnitude of any risk reduction may range from appreciable (i.e., RR < 0.75) to negligible. This uncertainty may be attributable to the heterogeneous interventions used, and the likelihood that some effects may vary by environment, population, probiotic strain, and prebiotic type. To address these issues, more high-quality, large, randomized controlled trials are needed. These studies should incorporate best practice methodologies [[72], [73], [74]], include diagnosis of GTI, and (when possible) identify specific pathogens causing infection. Within that context, confirmatory studies of specific probiotic strains and mixtures, prebiotic types, and synbiotic formulations will be especially critical to generate the evidence needed to tailor recommendations for particular interventions (i.e., specific probiotic strains or mixtures or prebiotic types) to specific populations and environmental exposures.

## Data Availability

The data generated during this study will be made available on a reasonable request.

## Appendix A Supplementary data

Multimedia component1 Multimedia component1
