# Supplementary material for: Orally Ingested Probiotic, Prebiotic, and Synbiotic Interventions as Countermeasures for Gastrointestinal Tract Infections in Nonelderly Adults: A Systematic Review and Meta-Analysis
Source: Adv Nutr. 2023 Feb 22;14(3):539–54. doi: 10.1016/j.advnut.2023.02.002 (PMC10201658; doi:10.1016/j.advnut.2023.02.002)
Supplement: Multimedia component1 [file mmc1.pdf]

Supplemental Table 1. PubMed search strategy.

(campylobacter*[TIAB] OR helicobacter*[TIAB] OR salmonell*[TIAB] OR shigella[TIAB] OR “clostridium difficile”[TIAB] OR “clostridioides difficile”[TIAB] OR clostridi*[TIAB] OR “peptoclostridium difficile”[TIAB] OR “escherichia coli”[TIAB] OR Escherichia*[TIAB] OR “e. coli”[TIAB] OR vibrio*[TIAB] OR yersini*[TIAB] OR plesiomonas[TIAB] OR aeromonas[TIAB] OR toxoplasm*[TIAB] OR rotavir*[TIAB] OR norovir*[TIAB] OR norwalk[TIAB] OR adenovir*[TIAB] OR astrovir*[TIAB] OR sapovir*[TIAB] OR entamoeb*[TIAB] OR cyclospor*[TIAB] OR cryptosporidi*[TIAB] OR giardia*[TIAB] OR “grimontia hollisae”[TIAB] OR balantidi***[TIAB]** OR dientamoeb***[TIAB]** OR diarrhea***[TIAB]** OR diarrhoea***[TIAB]** OR Gastroenteritis**[TIAB]** OR "gastrointestinal illness*"**[TIAB]** OR "gastrointestinal infection*"**[TIAB]** OR Enteritis**[TIAB]** OR "enteric infection*"**[TIAB]** OR "intestinal infection*”**[TIAB]** OR "infectious gastroenteritis”**[TIAB]** OR "traveler's diarrhea"**[TIAB]** OR "traveler's diarrhoea"**[TIAB]** OR “travelers’ diarrhea” **[TIAB]** OR “travelers’ diarrhoea” **[TIAB]** OR "traveller's diarrhea"**[TIAB]** OR "traveller's diarrhoea"**[TIAB]** OR “travellers’ diarrhea” **[TIAB]** OR “travellers’ diarrhoea” **[TIAB]** OR “travellers diarrhea” **[TIAB]** OR “travellers diarrhoea” **[TIAB]** OR cholera***[TIAB]** OR Dysentery**[TIAB]** OR Duodenitis**[TIAB]** OR Ileitis**[TIAB]** OR Enterocolitis**[TIAB]** OR Gastritis**[TIAB]** OR paratyphoid***[TIAB]** OR typhoid***[TIAB]** OR Campylobacter[MeSH] OR Helicobacter[Mesh:NoExp] OR Salmonella[Mesh:NoExp] OR Salmonella enterica[Mesh] OR "Shigella"[Mesh] OR Clostridium difficile[Mesh] OR Escherichia coli[Mesh:NoExp] OR Enteropathogenic Escherichia coli[Mesh] OR Enterotoxigenic Escherichia coli[Mesh] OR Escherichia coli K12[Mesh] OR Vibrio[Mesh:NoExp] OR Vibrio cholerae[Mesh] OR Vibrio parahaemolyticus[Mesh] OR Vibrio vulnificus[Mesh] OR Yersinia[Mesh:NoExp] OR Yersinia enterocolitica[Mesh] OR Plesiomonas[Mesh] OR Toxoplasma[Mesh] OR Aeromonas[Mesh:NoExp] OR Aeromonas caviae[Mesh] OR Aeromonas hydrophila[Mesh] OR Aeromonas veronii[Mesh] OR Rotavirus[Mesh] OR Norovirus[Mesh] OR Adenoviridae[Mesh:NoExp] OR Mastadenovirus[Mesh:NoExp] OR Adenoviruses, Human[Mesh] OR Astroviridae[Mesh:NoExp] OR Sapovirus[Mesh] OR Entamoeba[Mesh] OR Cyclospora[Mesh] OR Cryptosporidium[Mesh] OR Giardia[Mesh] OR Toxoplasmosis[Mesh:NoExp] OR Diarrhea[Mesh:NoExp] OR Gastroenteritis[Mesh:NoExp] OR Cholera Morbus[Mesh] OR Dysentery[Mesh] OR Enteritis[Mesh:NoExp] OR Duodenitis[Mesh] OR Ileitis[Mesh:NoExp] OR Enterocolitis[Mesh:NoExp] OR Gastritis[Mesh:NoExp] OR Gastrointestinal Diseases[Mesh:NoExp] OR Campylobacter Infections[Mesh] OR Helicobacter Infections[Mesh] OR Salmonella Infections[Mesh:NoExp] OR Paratyphoid Fever[Mesh] OR Salmonella Food Poisoning[Mesh] OR Typhoid Fever[Mesh] OR Clostridium Infections[Mesh:NoExp] OR Escherichia coli Infections[Mesh:NoExp] OR Vibrio Infections[Mesh] OR Yersinia Infections[Mesh:NoExp] OR Rotavirus Infections[Mesh] OR Adenoviridae Infections[Mesh:NoExp] OR Adenovirus Infections, Human[Mesh] OR Astroviridae Infections[Mesh] OR Entamoebiasis[Mesh] OR Cyclosporiasis[Mesh] OR Cryptosporidiosis[Mesh] OR Giardiasis[Mesh] OR Intestinal Diseases, Parasitic[Mesh:NoExp] OR Balantidiasis[Mesh] OR Dientamoebiasis[Mesh])
AND
(Probiotic*[TIAB] OR Lactobacill*[TIAB] OR Bifidobacteri*[TIAB] OR Enterococc*[TIAB] OR Lactococc*[TIAB] OR Saccharomyces[TIAB] OR “Streptococcus salivarius”[TIAB] OR “Streptococcus thermophiles”[TIAB] OR “Streptococcus faecalis”[TIAB] OR “Streptococcus

faecium"[TIAB] OR "Bacillus cereus"[TIAB] OR "Bacillus pumilus"[TIAB] OR "Bacillus coagulans"[TIAB] OR "Bacillus clausii"[TIAB] OR "Bacillus licheniformis"[TIAB] OR "Bacillus subtilis"[TIAB] OR "Bacillus indicus"[TIAB] OR "Bacillus mesentericus"[TIAB] OR "Bacillus polyfermenticus"[TIAB] OR "Bacillus laterosporus"[TIAB] OR "Bacillus polymyxa"[TIAB] OR "Clostridium butyricum"[TIAB] OR "Leuconostoc mesenteroides"[TIAB] OR *"Kluyveromyces marxianus"*[TIAB] OR "Pediococcus pentosaceus"[TIAB] OR "Pediococcus acidilacti"[TIAB] OR *"Propionibacterium freudenreichii*"[TIAB] OR Nissle[TIAB] OR lactis[TIAB] OR *delbrueckii*[TIAB] OR Kefir[TIAB] OR Yogurt[TIAB] OR "fermented milk"[TIAB] OR Florastor[TIAB] OR Bioflorin[TIAB] OR Culturelle[TIAB] OR VSL#3[TIAB] OR "Soluble fiber"[TIAB] OR "Soluble fibre"[TIAB] OR Psyllium[TIAB] OR "Fermentable fiber"[TIAB] OR "Fermentable fibre"[TIAB] OR "Fermentable polysaccharide*"[TIAB] OR "Fermentable oligosaccharide*"[TIAB] OR "Fermentable carbohydrate*"[TIAB] OR Prebiotic*[TIAB] OR Galactan*[TIAB] OR Fructan*[TIAB] OR Inulin[TIAB] OR Inulin-type[TIAB] OR "chicory root"[TIAB] OR Fructooligosaccharide*[TIAB] OR fructo-oligosaccharide*[TIAB] OR oligofructose[TIAB] OR galacto-oligosaccharide*[TIAB] OR galactooligosaccharide*[TIAB] OR bimuno[TIAB] OR lactulose[TIAB] OR kristalose[TIAB] OR enulose[TIAB] OR constulose[TIAB] OR "resistant starch"[TIAB] OR "high amylose starch"[TIAB] OR "high amylose maize"[TIAB] OR High-maize[TIAB] OR "high maize"[TIAB] OR "soluble corn fiber"[TIAB] OR Polydextrose[TIAB] OR litesse[TIAB] OR Arabinoxylan*[TIAB] OR "Arabinoxylan-oligosaccharide*"[TIAB] OR "Arabinoxylan oligosaccharide*"[TIAB] OR "milk oligosaccharide*"[TIAB] OR "Milk-oligosaccharide*"[TIAB] OR "2'-fucosyllactose" [TIAB] OR "Lacto-*N*-neotetraose" [TIAB] OR "xylo-oligosaccharide*"[TIAB] OR xylooligosaccharide*[TIAB] OR isomalto-oligosaccharide*[TIAB] OR isomaltooligosaccharide*[TIAB] OR "soy oligosaccharide*"[TIAB] OR Soy-oligosaccharide*[TIAB] OR Raffinose[TIAB] OR stachyose[TIAB] OR mannan-oligosaccharide*[TIAB] OR mannanoligosaccharide*[TIAB] OR pectic-oligosaccharide*[TIAB] OR pecticoligosaccharide*[TIAB] OR "limit dextrin*"[TIAB] OR Benefiber[TIAB] OR "Resistant maltodextrin*"[TIAB] OR Pectin*[TIAB] OR beta-glucan*[TIAB] OR β-glucan*[TIAB] OR beta glucan*[TIAB] OR "guar gum" [TIAB] OR "locust bean gum"[TIAB] OR "carob gum" [TIAB] OR Carobin[TIAB] OR "Fenugreek gum"[TIAB] OR "acacia gum*"[TIAB] OR "gum Arabic"[TIAB] OR "gum sudani"[TIAB] OR "Arabic gum*"[TIAB] OR "senegal gum*"[TIAB] OR "indian gum*"[TIAB] OR beta-glycan*[TIAB] OR β-glycan*[TIAB] OR glucomannan*[TIAB] OR isphaghula[TIAB] OR lactosucrose[TIAB] OR Alginate*[TIAB] OR Synbiotic*[TIAB] OR Symbiotic*[TIAB] OR Probiotics[Mesh] OR Lactobacillus[Mesh:NoExp] OR Lactobacillus acidophilus[Mesh] OR Lactobacillus brevis[Mesh] OR Lactobacillus casei[Mesh] OR Lactobacillus crispatus[Mesh] OR Lactobacillus delbrueckii[Mesh] OR Lactobacillus fermentum[Mesh] OR Lactobacillus gasseri[Mesh] OR Lactobacillus helveticus[Mesh] OR Lactobacillus johnsonii[Mesh] OR Lactobacillus paracasei[Mesh] OR Lactobacillus pentosus[Mesh] OR Lactobacillus plantarum[Mesh] OR Lactobacillus reuteri[Mesh] OR Lactobacillus rhamnosus[Mesh] OR Lactobacillus sakei[Mesh] OR Lactobacillus salivarius[Mesh] OR Bifidobacterium[Mesh] OR Streptococcus salivarius[Mesh] OR Streptococcus thermophilus[Mesh] OR Bacillus clausii[Mesh] OR Bacillus coagulans[Mesh] OR Bacillus licheniformis[Mesh] OR Bacillus subtilis[Mesh] OR Saccharomyces[Mesh:NoExp] OR Saccharomyces boulardii[Mesh] OR

Lactococcus[Mesh] OR Enterococcus[Mesh:NoExp] OR Enterococcus faecalis[Mesh] OR Enterococcus faecium[Mesh] OR Kefir[Mesh] OR Yogurt[Mesh] OR Carrageenan[Mesh] OR Chitin[Mesh] OR Fructans[Mesh] OR Galactans[Mesh:NoExp] OR Glucans[Mesh:NoExp] OR beta-Glucans[Mesh:NoExp] OR Lentinan[Mesh] OR Sizofiran[Mesh] OR Lactulose[Mesh] OR Raffinose[Mesh] OR Pectins[Mesh] OR Plant Gums[Mesh:NoExp] OR Gum Arabic[Mesh] OR Karaya Gum[Mesh] OR Tragacanth[Mesh] OR Plant Mucilage[Mesh] OR Prebiotics[Mesh] OR Synbiotics[Mesh])
AND
("randomized controlled trial"[Publication Type] OR "controlled clinical trial"[Publication Type] OR "randomized"[TIAB] OR "placebo"[TIAB] OR "drug therapy"[MeSH Subheading] OR "randomly"[TIAB] OR "trial"[TIAB] OR "groups"[TIAB])

Supplemental Table 2. Web of Science search strategy.

TI=**(**campylobacter* OR helicobacter* OR salmonell* OR Shigella OR "Clostridium difficile" OR clostridi* OR "clostridioides difficile" OR "peptoclostridium difficile" OR "Escherichia coli" OR escherichia* OR "e. coli" OR vibrio* OR yersini* OR Plesiomonas OR Aeromonas OR toxoplasm* OR rotavir* OR norovir* OR Norwalk OR adenovir* OR astrovir* OR sapovir* OR entamoeb* OR cyclospor* OR cryptosporidi* OR giardia* OR "grimontia hollisae" OR balantidi* OR dientamoeb* OR diarrhea* OR diarrhoea* OR Gastroenteritis OR "gastrointestinal illness*" OR "gastrointestinal infection*" OR Enteritis OR "enteric infection*" OR "intestinal infection*" OR "infectious gastroenteritis" OR "traveler's diarrhea" OR "traveler's diarrhoea" OR "travelers' diarrhea" OR "travelers' diarrhoea" OR "traveller's diarrhea" OR "traveller's diarrhoea" OR "travellers' diarrhea" OR "travellers' diarrhoea" OR "travellers diarrhea" OR "travellers diarrhoea" OR cholera* OR Dysentery OR Duodenitis OR Ileitis OR Enterocolitis OR Gastritis OR paratyphoid* OR typhoid*) OR AB=(campylobacter* OR helicobacter* OR salmonell* OR Shigella OR "Clostridium difficile" OR clostridi* OR "clostridioides difficile" OR "peptoclostridium difficile" OR "Escherichia coli" OR escherichia* OR "e. coli" OR vibrio* OR yersini* OR Plesiomonas OR Aeromonas OR toxoplasm* OR rotavir* OR norovir* OR Norwalk OR adenovir* OR astrovir* OR sapovir* OR entamoeb* OR cyclospor* OR cryptosporidi* OR giardia* OR "grimontia hollisae" OR balantidi* OR dientamoeb* OR diarrhea* OR diarrhoea* OR Gastroenteritis OR "gastrointestinal illness*" OR "gastrointestinal infection*" OR Enteritis OR "enteric infection*" OR "intestinal infection*" OR "infectious gastroenteritis" OR "traveler's diarrhea" OR "traveler's diarrhoea" OR "travelers' diarrhea" OR "travelers' diarrhoea" OR "traveller's diarrhea" OR "traveller's diarrhoea" OR "travellers' diarrhea" OR "travellers' diarrhoea" OR "travellers diarrhea" OR "travellers diarrhoea" OR cholera* OR Dysentery OR Duodenitis OR Ileitis OR Enterocolitis OR Gastritis OR paratyphoid* OR typhoid*)<br>AND<br>TI=(probiotic* OR lactobacill* OR bifidobacteri* OR enterococc* OR lactococc* OR Saccharomyces OR "streptococcus salivarius" OR " streptococcus thermophilus" OR "streptococcus faecalis" OR "streptococcus faecium" OR "bacillus cereus" OR **"**Bacillus pumilus" OR "Bacillus coagulans" OR "Bacillus clausii" OR "Bacillus licheniformis" OR "Bacillus subtilis" OR "Bacillus indicus" OR "Bacillus mesentericus" OR "Bacillus polyfermenticus" OR "Bacillus laterosporus" OR "Bacillus polymyxa" OR "Clostridium butyricum" OR "Leuconostoc mesenteroides" OR *"Kluyveromyces marxianus" OR* "Pediococcus pentosaceus" OR "Pediococcus acidilacti" OR *"Propionibacterium freudenreichii"* OR Nissle OR lactis OR *delbrueckii OR* Kefir OR Yogurt OR "fermented milk" OR Florastor OR Bioflorin OR Culturelle OR "Soluble fiber" OR "Soluble fibre" OR Psyllium OR "Fermentable fiber" OR "Fermentable fibre" OR "Fermentable polysaccharide*" OR "Fermentable oligosaccharide*" OR "Fermentable carbohydrate*" OR Prebiotic* OR Galactan* OR Fructan* OR Inulin OR Inulin-type OR "chicory root" OR Fructooligosaccharide* OR fructo-oligosaccharide* OR oligofructose OR galacto-oligosaccharide* OR galactooligosaccharide* OR bimuno OR lactulose OR kristalose OR enulose OR constulose OR "resistant starch" OR "high amylose starch" OR "high amylose maize" OR High-maize OR "high maize" OR "soluble corn fiber" OR Polydextrose OR litesse OR Arabinoxylan* OR Arabinoxylan-oligosaccharide* OR "Arabinoxylan oligosaccharide*" OR "milk oligosaccharide*" OR milk-oligosaccharide* OR 2'-fucosyllactose OR Lacto-N-

neotetraose OR xylo-oligosaccharide* OR xylooligosaccharide* OR isomalto-oligosaccharide* OR isomaltooligosaccharide* OR "soy oligosaccharide*" OR Soy-oligosaccharide* OR Raffinose OR stachyose OR mannan-oligosaccharide* OR mannanoligosaccharide* OR pectic-oligosaccharide* OR pecticoligosaccharide* OR "limit dextrin*" OR Benefiber OR "Resistant maltodextrin*" OR Pectin* OR beta-glucan* OR β-glucan* OR "beta glucan*" OR "guar gum" OR "locust bean gum" OR "carob gum" OR Carobin OR "Fenugreek gum" OR "acacia gum*" OR "gum Arabic" OR "gum sudani" OR "Arabic gum*" OR "senegal gum*" OR "indian gum*" OR beta-glycan* OR β-glycan* OR glucomannan* OR ispaghula OR lactosucrose OR Alginate* OR Synbiotic* OR Symbiotic*) OR AB=**(**probiotic* OR lactobacill* OR bifidobacteri* OR enterococc* OR lactococc* OR Saccharomyces OR "streptococcus salivarius" OR " streptococcus thermophilus" OR "streptococcus faecalis" OR "streptococcus faecium" OR "bacillus cereus" OR "Bacillus pumilus" OR "Bacillus coagulans" OR "Bacillus clausii" OR "Bacillus licheniformis" OR "Bacillus subtilis" OR "Bacillus indicus" OR "Bacillus mesentericus" OR "Bacillus polyfermenticus" OR "Bacillus laterosporus" OR "Bacillus polymyxa" OR "Clostridium butyricum" OR "Leuconostoc mesenteroides" OR *"Kluyveromyces marxianus" OR* "Pediococcus pentosaceus" OR "Pediococcus acidilacti" OR *"Propionibacterium freudenreichii"* OR Nissle OR lactis OR *delbrueckii OR* Kefir OR Yogurt OR "fermented milk" OR Florastor OR Bioflorin OR Culturelle OR "Soluble fiber" OR "Soluble fibre" OR Psyllium OR "Fermentable fiber" OR "Fermentable fibre" OR "Fermentable polysaccharide*" OR "Fermentable oligosaccharide*" OR "Fermentable carbohydrate*" OR Prebiotic* OR Galactan* OR Fructan* OR Inulin OR Inulin-type OR "chicory root" OR Fructooligosaccharide* OR fructo-oligosaccharide* OR oligofructose OR galacto-oligosaccharide* OR galactooligosaccharide* OR bimuno OR lactulose OR kristalose OR enulose OR constulose OR "resistant starch" OR "high amylose starch" OR "high amylose maize" OR High-maize OR "high maize" OR "soluble corn fiber" OR Polydextrose OR litesse OR Arabinoxylan* OR Arabinoxylan-oligosaccharide* OR "Arabinoxylan oligosaccharide*" OR "milk oligosaccharide*" OR milk-oligosaccharide* OR 2'-fucosyllactose OR Lacto-N-neotetraose OR xylo-oligosaccharide* OR xylooligosaccharide* OR isomalto-oligosaccharide* OR isomaltooligosaccharide* OR "soy oligosaccharide*" OR Soy-oligosaccharide* OR Raffinose OR stachyose OR mannan-oligosaccharide* OR mannanoligosaccharide* OR pectic-oligosaccharide* OR pecticoligosaccharide* OR "limit dextrin*" OR Benefiber OR "Resistant maltodextrin*" OR Pectin* OR beta-glucan* OR β-glucan* OR "beta glucan*" OR "guar gum" OR "locust bean gum" OR "carob gum" OR Carobin OR "Fenugreek gum" OR "acacia gum*" OR "gum Arabic" OR "gum sudani" OR "Arabic gum*" OR "senegal gum*" OR "indian gum*" OR beta-glycan* OR β-glycan* OR glucomannan* OR ispaghula OR lactosucrose OR Alginate* OR Synbiotic* OR Symbiotic*)
AND
TS=(randomised OR randomized OR randomisation OR randomisation OR placebo* OR (random* AND (allocat* OR assign*)) OR (blind* AND (single OR double OR treble OR triple)))

Supplemental Table 3. Scopus search strategy.

(INDEXTERMS ("clinical trials" OR "clinical trials as a topic" OR "randomized controlled trial" OR "Randomized Controlled Trials as Topic" OR "controlled clinical trial" OR "Controlled Clinical Trials" OR "random allocation" OR "Double-Blind Method" OR "Single-Blind Method" OR "Cross-Over Studies" OR "Placebos" OR "multicenter study" OR "double blind procedure" OR "single blind procedure" OR "crossover procedure" OR "clinical trial" OR "controlled study" OR "randomization" OR "placebo") OR TITLE-ABS-KEY ("clinical trials" OR "clinical trials as a topic" OR "randomized controlled trial" OR "Randomized Controlled Trials as Topic" OR "controlled clinical trial" OR "Controlled Clinical Trials as Topic" OR "random allocation" OR "randomly allocated" OR "allocated randomly" OR "Double-Blind Method" OR "Single-Blind Method" OR "Cross-Over Studies" OR "Placebos" OR "cross-over trial" OR "single blind" OR "double blind" OR "factorial design" OR "factorial trial") OR TITLE-ABS (clinical AND trial* OR trial* OR rct* OR random* OR blind*)) AND (TITLE-ABS (probiotic* OR lactobacill* OR bifidobacteri* OR enterococc* OR lactococc* OR saccharomyces OR "streptococcus salivarius" OR " streptococcus thermophilus" OR "streptococcus faecalis" OR "streptococcus faecium" OR "bacillus cereus" OR "Bacillus pumilus" OR "Bacillus coagulans" OR "Bacillus clausii" OR "Bacillus licheniformis" OR "Bacillus subtilis" OR "Bacillus indicus" OR "Bacillus mesentericus" OR "Bacillus polyfermenticus" OR "Bacillus laterosporus" OR "Bacillus polymyxa" OR "Clostridium butyricum" OR "Leuconostoc mesenteroides" OR "Kluyveromyces marxianus" OR "Pediococcus pentosaceus" OR "Pediococcus acidilacti" OR "Propionibacterium freudenreichii" OR nissle OR lactis OR delbrueckii OR kefir OR yogurt OR "fermented milk" OR florastor OR bioflorin OR culturelle OR vsl#3 OR "Soluble fiber" OR "Soluble fibre" OR psyllium OR "Fermentable fiber" OR "Fermentable fibre" OR "Fermentable polysaccharide*" OR "Fermentable oligosaccharide*" OR "Fermentable carbohydrate*" OR prebiotic* OR galactan* OR fructan* OR inulin OR inulin-type OR "chicory root" OR fructooligosaccharide* OR fructo-oligosaccharide* OR oligofructose OR galacto-oligosaccharide* OR galactooligosaccharide* OR bimuno OR lactulose OR kristalose OR enulose OR constulose OR "resistant starch" OR "high amylose starch" OR "high amylose maize" OR high-maize OR "high maize" OR "soluble corn fiber" OR polydextrose OR litesse OR arabinoxylan* OR arabinoxylan-oligosaccharide* OR "Arabinoxylan oligosaccharide*" OR "milk oligosaccharide*" OR milk-oligosaccharide* OR 2'-fucosyllactose OR lacto-n-neotetraose OR xylo-oligosaccharide* OR xylooligosaccharide* OR isomalto-oligosaccharide* OR isomaltooligosaccharide* OR "soy oligosaccharide*" OR soy-

oligosaccharide* OR raffinose OR stachyose OR mannan-oligosaccharide* OR mannanoligosaccharide* OR pectic-oligosaccharide* OR pecticoligosaccharide* OR "limit dextrin*" OR benefiber OR "Resistant maltodextrin*" OR pectin* OR beta-glucan* OR β-glucan* OR "beta glucan*" OR "guar gum" OR "locust bean gum" OR "carob gum" OR carobin OR "Fenugreek gum" OR "acacia gum*" OR "gum Arabic" OR "gum sudani" OR "Arabic gum*" OR "senegal gum*" OR "indian gum*" OR beta-glycan* OR β-glycan* OR glucomannan* OR ispaghula OR lactosucrose OR alginate* OR synbiotic* OR symbiotic*)) AND (TITLE-ABS (campylobacter* OR helicobacter* OR salmonell* OR shigella OR "Clostridium difficile" OR clostridi* OR "clostridioides difficile" OR "peptoclostridium difficile" OR "Escherichia coli" OR escherichia* OR "e. coli" OR vibrio* OR yersini* OR plesiomonas OR aeromonas OR toxoplasm* OR rotavir* OR norovir* OR norwalk OR adenovir* OR astrovir* OR sapovir* OR entamoeb* OR cyclospor* OR cryptosporidi* OR giardia* OR "grimontia hollisae" OR balantidi* OR dientamoeb* OR diarrhea* OR diarrhoea* OR gastroenteritis OR "gastrointestinal illness*" OR "gastrointestinal infection*" OR enteritis OR "enteric infection*" OR "intestinal infection*" OR "infectious gastroenteritis" OR "traveler's diarrhea" OR "traveler's diarrhoea" OR "travelers' diarrhea" OR "travelers' diarrhoea" OR "traveller's diarrhea" OR "traveller's diarrhoea" OR "travellers' diarrhea" OR "travellers' diarrhoea" OR "travellers diarrhea" OR "travellers diarrhoea" OR cholera* OR dysentery OR duodenitis OR ileitis OR enterocolitis OR gastritis OR paratyphoid* OR typhoid*)) AND (LIMIT-TO (LANGUAGE , "English"))

Supplemental Table 4. CENTRAL search strategy.

campylobacter* OR helicobacter* OR salmonell* OR Shigella OR "Clostridium difficile" OR clostridi* OR “clostridioides difficile" OR "peptoclostridium difficile" OR "Escherichia coli" OR escherichia* OR "e. coli" OR vibrio* OR yersini* OR Plesiomonas OR Aeromonas OR toxoplasm* OR rotavir* OR norovir* OR Norwalk OR adenovir* OR astrovir* OR sapovir* OR entamoeb* OR cyclospor* OR cryptosporidi* OR giardia* OR "grimontia hollisae" OR balantidi* OR dientamoeb* OR diarrhea* OR diarrhoea* OR Gastroenteritis OR "gastrointestinal illness*" OR "gastrointestinal infection*" OR Enteritis OR "enteric infection*" OR "intestinal infection*” OR "infectious gastroenteritis" OR "traveler's diarrhea" OR "traveler's diarrhoea" OR “travelers’ diarrhea” OR “travelers’ diarrhoea” OR "traveller's diarrhea" OR "traveller's diarrhoea" OR “travellers’ diarrhea” OR “travellers’ diarrhoea” OR “travellers diarrhea” OR “travellers diarrhoea” OR cholera* OR Dysentery OR Duodenitis OR Ileitis OR Enterocolitis OR Gastritis OR paratyphoid* OR typhoid*

AND

probiotic* OR lactobacill* OR bifidobacteri* OR enterococc* OR lactococc* OR Saccharomyces OR “streptococcus salivarius” OR “streptococcus thermophilus” OR “streptococcus faecalis” OR “streptococcus faecium” OR “bacillus cereus” OR “Bacillus pumilus” OR “Bacillus coagulans” OR “Bacillus clausii” OR “Bacillus licheniformis” OR “Bacillus subtilis” OR “Bacillus indicus” OR “Bacillus mesentericus” OR “Bacillus polyfermenticus” OR “Bacillus laterosporus” OR “Bacillus polymyxa” OR “Clostridium butyricum” OR “Leuconostoc mesenteroides” OR *“Kluyveromyces marxianus” OR* “Pediococcus pentosaceus” OR “Pediococcus acidilacti” OR *“Propionibacterium freudenreichii”* OR Nissle OR lactis OR *delbrueckii OR* Kefir OR Yogurt OR “fermented milk” OR Florastor OR Bioflorin OR Culturelle OR “Soluble fiber” OR “Soluble fibre” OR Psyllium OR “Fermentable fiber” OR “Fermentable fibre” OR “Fermentable polysaccharide*” OR “Fermentable oligosaccharide*” OR “Fermentable carbohydrate*” OR Prebiotic* OR Galactan* OR Fructan* OR Inulin OR Inulin-type OR “chicory root” OR Fructooligosaccharide* OR fructo-oligosaccharide* OR oligofructose OR galacto-oligosaccharide* OR galactooligosaccharide* OR bimuno OR lactulose OR kristalose OR enulose OR constulose OR “resistant starch” OR “high amylose starch” OR “high amylose maize” OR High-maize OR “high maize” OR “soluble corn fiber” OR Polydextrose OR litesse OR Arabinoxylan* OR Arabinoxylan-oligosaccharide* OR “Arabinoxylan oligosaccharide*” OR "milk oligosaccharide*" OR milk-oligosaccharide* OR fucosyllactose OR Lacto-N-neotetraose OR xylo-oligosaccharide* OR xylooligosaccharide* OR isomalto-oligosaccharide* OR isomaltooligosaccharide* OR “soy oligosaccharide*” OR Soy-oligosaccharide* OR Raffinose OR stachyose OR mannan-oligosaccharide* OR mannanoligosaccharide* OR pectic-oligosaccharide* OR pecticoligosaccharide* OR “limit dextrin*” OR Benefiber OR “Resistant maltodextrin*” OR Pectin* OR beta-glucan* OR β-glucan* OR “beta glucan*” OR “guar gum” OR “locust bean gum” OR “carob gum” OR Carobin OR “Fenugreek gum” OR “acacia gum*” OR “gum Arabic” OR “gum sudani” OR “Arabic gum*” OR “senegal gum*” OR “indian gum*” OR beta-glycan* OR b-glycan* OR glucomannan* OR ispaghula OR lactosucrose OR Alginate* OR Synbiotic* OR Symbiotic*

Supplemental Table 5. Probiotics and prebiotics used as interventions in included studies.

	Lactobacillus									***Bifidobacterium***						**Prebiotic**	
	acidophilus	***bulgaricus***	***casei***	***fermentum***	***gasseri***	***helveticus* Rosell-52**	***plantarum***	***reuteri***	***rhamnosus***	***animalis ssp. lactis***	***bifidum***	***longum***	***S. thermophilus***	***Sa. cerevisiae***	***E. faecium* SF68**	***FOS***	**Bimuno-GOS**
Probiotic, single strain																	
Hilton 1997									X[2]								
Kollaritsch 1989_Study 4														X[4]			
Kollaritsch 1989_Study 1	X																
Liu 2020							X[5]										
Oksanen 1990									X[2]								
Ouwenhand 2014	X[6]																
Pereg 2005			X[1]														
Schroder 2015								X									
Probiotic, multi-strain																	
Guillemard 2010		X	X[1]										X				
Haywood 2014					X						X	X					
Kalima 2016									X[2]	X[3]							
Pozo-Olano 1978	X	X															
Pumpa 2019[7]	X		X	X			X		X	X	X		X	X			
Ten Bruggencate 2015						X			X[8]			X[9]		X[10]			
Prebiotic																	
Cummings 2001																X	
Drakoularakou 2010																	X
Hasle 2017																	X
Synbiotic																	
Virk 2013														X[11]	X	X	

E.: *Enterococcus*; FOS: fructooligosaccharide; GOS: galactooligosaccharide; S.: *Streptococcus*; Sa.: *Saccharomyces.*
[1]strain: DN-114-001
[2]strain: GG
[3]strain: BB12
[4]strain: Hansen CBS 5926
[5]strain: DR7
[6]strain: ATCC 700396
[7]product: UltraBiotic 60 (BioCeuticals; New South Wales, Australia)
[8]strain: Rosell-11
[9]strain: Rosell-175
[10]strain: CNCM I-1079
[11]strain: CNCM I-4444

References:

Cummings JH, Christie S, Cole TJ. A study of fructo oligosaccharides in the prevention of travellers' diarrhoea. Alimentary pharmacology & therapeutics 2001;15(8):1139-45. doi: 10.1046/j.1365-2036.2001.01043.x.

de dios Pozo-Olano J, Warram JH, Jr., Gómez RG, Cavazos MG. Effect of a lactobacilli preparation on traveler's diarrhea. A randomized, double blind clinical trial. Gastroenterology 1978;74(5 Pt 1):829-30.

Drakoularakou A, Tzortzis G, Rastall RA, Gibson GR. A double-blind, placebo-controlled, randomized human study assessing the capacity of a novel galacto-oligosaccharide mixture in reducing travellers' diarrhoea. European journal of clinical nutrition 2010;64(2):146-52. doi: 10.1038/ejcn.2009.120.

Guillemard E, Tanguy J, Flavigny A, de la Motte S, Schrezenmeir J. Effects of consumption of a fermented dairy product containing the probiotic Lactobacillus casei DN-114 001 on common respiratory and gastrointestinal infections in shift workers in a randomized controlled trial. Journal of the American College of Nutrition 2010;29(5):455-68. doi: 10.1080/07315724.2010.10719882.

Hasle G, Raastad R, Bjune G, Jenum PA, Heier L. Can a galacto-oligosaccharide reduce the risk of traveller's diarrhoea? A placebo-controlled, randomized, double-blind study. Journal of travel medicine 2017;24(5). doi: 10.1093/jtm/tax057.Haywood BA, Black KE, Baker D, McGarvey J, Healey P, Brown RC. Probiotic supplementation reduces the duration and incidence of infections but not severity in elite rugby union players. Journal of science and medicine in sport 2014;17(4):356-60. doi: 10.1016/j.jsams.2013.08.004.

Haywood BA, Black KE, Baker D, McGarvey J, Healey P, Brown RC. Probiotic supplementation reduces the duration and incidence of infections but not severity in elite rugby union players. Journal of science and medicine in sport 2014;17(4):356-60. doi: 10.1016/j.jsams.2013.08.004.

Hilton E, Kolakowski P, Singer C, Smith M. Efficacy of Lactobacillus GG as a Diarrheal Preventive in Travelers. Journal of travel medicine 1997;4(1):41-3. doi: 10.1111/j.1708-8305.1997.tb00772.x.

Kalima K, Lehtoranta L, He L, Pitkäniemi J, Lundell R, Julkunen I, Roivainen M, Närkiö M, Mäkelä MJ, Siitonen S, et al. Probiotics and respiratory and gastrointestinal tract infections in Finnish military conscripts - a randomised placebo-controlled double-blinded study. Beneficial microbes 2016;7(4):463-71. doi: 10.3920/bm2015.0172.

Kollaritsch H, Kremsner PG, Wiedermann G, Scheiner O. Prevention of traveller ' s diarrhea : Comparison of different non-antibiotic preparations. 1989:1-9.

Liu G, Chong HX, Chung FY, Li Y, Liong MT. Lactobacillus plantarum DR7 Modulated Bowel Movement and Gut Microbiota Associated with Dopamine and Serotonin Pathways in Stressed Adults. International journal of molecular sciences 2020;21(13). doi: 10.3390/ijms21134608.

Oksanen PJ, Salminen S, Saxelin M, Hämäläinen P, Ihantola-Vormisto A, Muurasniemi-Isoviita L, Nikkari S, Oksanen T, Pörsti I, Salminen E, et al. Prevention of travellers' diarrhoea by Lactobacillus GG. Annals of medicine 1990;22(1):53-6. doi: 10.3109/07853899009147242.

Ouwehand AC, ten Bruggencate SJ, Schonewille AJ, Alhoniemi E, Forssten SD, Bovee-Oudenhoven IM. Lactobacillus acidophilus supplementation in human subjects and their resistance to enterotoxigenic Escherichia coli infection. The British journal of nutrition 2014;111(3):465-73. doi: 10.1017/s0007114513002547.

Pereg D, Kimhi O, Tirosh A, Orr N, Kayouf R, Lishner M. The effect of fermented yogurt on the prevention of diarrhea in a healthy adult population. American journal of infection control 2005;33(2):122-5. doi: 10.1016/j.ajic.2004.11.001.

Pumpa KL, McKune AJ, Harnett J. A novel role of probiotics in improving host defence of elite rugby union athlete: A double blind randomised controlled trial. Journal of science and medicine in sport 2019;22(8):876-81. doi: 10.1016/j.jsams.2019.03.013.

Schröder C, Schmidt S, Garbe E, Röhmel J, Giersiepen K. Effects of the regular intake of the probiotic Lactobacillus reuteri (DSM 17938) on respiratory and gastrointestinal infections in a workplace setting: a double-blind randomized placebo-controlled trial. BMC Nutrition 2015;1(1):3. doi: 10.1186/2055-0928-1-3.

Ten Bruggencate SJ, Girard SA, Floris-Vollenbroek EG, Bhardwaj R, Tompkins TA. The effect of a multi-strain probiotic on the resistance toward Escherichia coli challenge in a randomized, placebo-controlled, double-blind intervention study. European journal of clinical nutrition 2015;69(3):385-91. doi: 10.1038/ejcn.2014.238.

Virk A, Mandrekar J, Berbari EF, Boyce TG, Fischer PR, Kasten MJ, Orenstein R, Rosenblatt JE, Sampathkumar P, Sia I, et al. A randomized, double blind, placebo-controlled trial of an oral synbiotic (AKSB) for prevention of travelers' diarrhea. Journal of travel medicine 2013;20(2):88-94. doi: 10.1111/jtm.12008.

Supplemental Figure 1

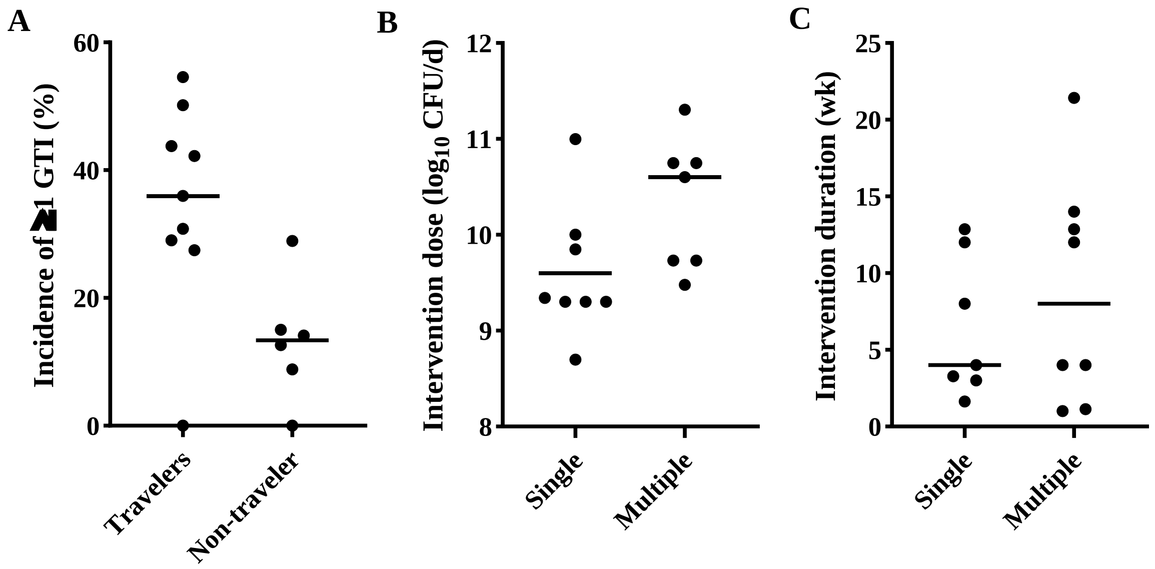

Supplemental Figure 1. Characteristics of studies identified in a systematic review of studies examining the effects of orally ingested probiotics versus placebo on the incidence and duration of gastrointestinal tract infections (GTIs) in non-elderly adults. **A)** Incidence proportion of complete case cohort experiencing one or more GTI during the intervention period. **B)** Daily dose of probiotic administered and **C)** duration of probiotic administration according to whether interventions included a single probiotic or multiple probiotic strains. **A-C)** Several studies identified by the search are not included in the plots due to not being applicable or to not reporting relevant data.

Supplemental Figure 2

A

Pop.	Intervention	Study name	Risk ratio	Lower limit	Upper limit	p-Value	GTI / Total -biotic	GTI / Total Placebo
T	L. rhamnosus GG	Oksanen 1990	0.89	0.76	1.06	0.19	153 / 402	178 / 418
NT	L. casei DN-114-01	Pereg 2015	0.75	0.48	1.16	0.20	31 / 275	40 / 266
NT	L. plantarum DR7	Liu 2020	0.75	0.28	2.04	0.57	6 / 62	8 / 62
NT	L. reuteri	Schroder 2015	0.53	0.31	0.93	0.03	16 / 121	30 / 121
T	Multi strain_1	Pozo-Olano 1978_Grp2	2.88	0.71	11.73	0.14	7 / 17	2 / 14
NT	Multi strain_2	Guillemard 2010	0.89	0.59	1.34	0.57	40 / 500	45 / 500
		Pooled	0.83	0.68	1.02	0.07		

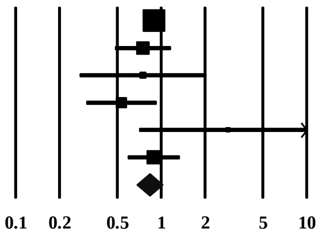

B

Pop.	Intervention	Study name	Risk ratio	Lower limit	Upper limit	p-Value	GTI / Total -biotic	GTI / Total Placebo
T	L. rhamnosus GG	Oksanen 1990	0.89	0.76	1.04	0.15	166 / 402	194 / 418
NT	L. casei DN-114-01	Pereg 2015	0.78	0.51	1.17	0.23	34 / 275	43 / 266
NT	L. plantarum DR7	Liu 2020	0.76	0.30	1.93	0.57	7 / 62	9 / 62
NT	L. reuteri	Schroder 2015	0.70	0.48	1.02	0.06	32 / 121	45 / 121
T	Multi strain_1	Pozo-Olano 1978_Grp2	2.88	0.71	11.73	0.14	7 / 17	2 / 14
NT	Multi strain_2	Guillemard 2010	0.90	0.61	1.35	0.62	42 / 500	46 / 500
		Pooled	0.86	0.76	0.98	0.02		

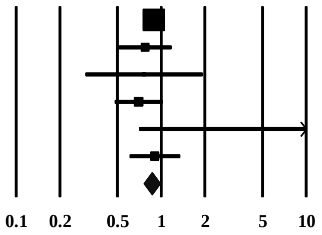

Supplemental Figure 2. Forest plots of the effects of orally ingested probiotics versus placebo on the incidence of gastrointestinal tract infections (GTI) in non-elderly adults. Intention-to-treat analysis **A)** assuming no cases among participants with missing data (I^2 = 22.0, p = 0.27), and **B)** assuming the risk ratio among participants with missing data matched the risk ratio of the control group complete case cohort (I^2 = 0, p = 0.47). Random effects meta-analysis using DerSimonian and Laird inverse variance method. Individual study effect estimates (squares; sized by study weight) and pooled effects (diamonds) are plotted. Lower and upper limits are 95% CIs. Grp, group; H., Hansen; L., *Lactobacillus*; NT, non-traveler; Pop., population; S., *Saccharomyces*; T, travelers. Multi-strain_1: *L. acidophilus*, *L. bulgaricus*; Multi-strain_2: *L. casei* DN-114-001, *Streptococcus thermophilus, L. delbreuckii.*

Supplemental Figure 3

Pop.	Intervention	Study name	Statistics for each study				Sample size	
			Difference in means	Lower limit	Upper limit	p-Value	-biotic	Placebo
NT	L. casei DN-114-01	Pereg 2015	0.24	-0.28	0.76	0.37	52	58
NT	L. plantarum DR7	Liu 2020	-0.03	-0.86	0.81	0.95	12	15
T	Multi strain_1	Pozo-Olano 1978_Grp2	-0.60	-3.61	2.41	0.70	7	2
		Pooled	0.15	-0.29	0.58	0.50		

Difference in means and 95% CI

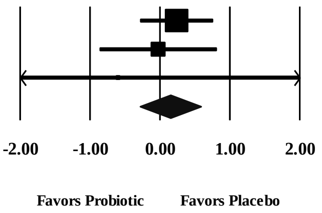

Supplemental Figure 3. Forest plot of the effects of orally ingested probiotics versus placebo on the duration of gastrointestinal tract infections (GTI) in non-elderly adults. Intention-to-treat analysis assuming the mean episode duration (days per GTI episode) among participants with missing data matched the mean duration of the control group complete case cohort ($I^2 = 0$, p = 0.77). Random effects meta-analysis using DerSimonian and Laird inverse variance method. Individual study effect estimates (squares; sized by study weight) and pooled effects (diamond) are plotted. Lower and upper limits are 95% CIs. Grp, group; L., *Lactobacillus*; NT, non-traveler; Pop., population; T, travelers. Multi-strain_1: *L. acidophilus*, *L. bulgaricus*.

Supplemental Figure 4

A

Pop.	Intervention	Study name	Rate ratio	Lower limit	Upper limit	p-Value	GTI / Total -biotic	GTI / Total Placebo
NT	L. plantarum DR7	Liu 2020	0.80	0.41	1.54	0.51	16 / 14	20 / 14
NT	L. reuteri	Schroder 2015	0.44	0.31	0.63	0.00	47 / 30	106 / 30
T	Multi strain_1	Pozo-Olano 1978_Grp2	2.33	0.92	5.91	0.07	17 / 0	6 / 0
NT	Multi strain_2	Guillemard 2010	1.00	0.80	1.25	1.00	150 / 115	150 / 115
NT	Multi strain_3	Kalima 2016_90d	1.55	1.29	1.87	0.00	278 / 50	185 / 52
NT	Multi strain_3	Kalima 2016_150d	0.67	0.51	0.88	0.00	88 / 117	131 / 117
		Pooled	0.93	0.60	1.43	0.73		

Rate ratio and 95% CI

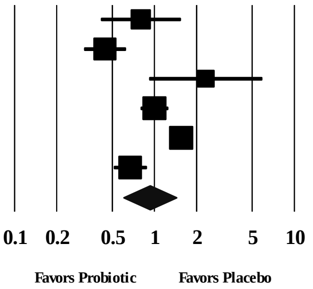

B

Pop.	Intervention	Study name	Rate ratio	Lower limit	Upper limit	p-Value	GTI / Total -biotic	GTI / Total Placebo
NT	L. plantarum DR7	Liu 2020	0.81	0.44	1.50	0.50	18 / 14	23 / 14
NT	L. reuteri	Schroder 2015	0.64	0.50	0.82	0.00	102 / 30	160 / 30
T	Multi strain_1	Pozo-Olano 1978_Grp2	2.33	0.92	5.91	0.07	17 / 0	6 / 0
NT	Multi strain_2	Guillemard 2010	1.01	0.81	1.26	0.92	157 / 115	155 / 115
NT	Multi strain_3	Kalima 2016_90d	1.17	1.02	1.34	0.03	427 / 50	379 / 52
NT	Multi strain_3	Kalima 2016_150d	0.95	0.84	1.08	0.43	484 / 117	510 / 117
		Pooled	0.96	0.78	1.17	0.69		

Rate ratio and 95% CI

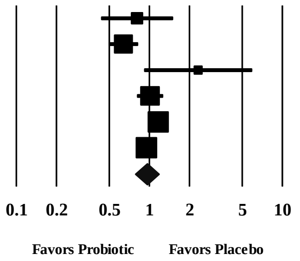

Supplemental Figure 4. Forest plots pf the effects of orally ingested probiotics versus placebo on the rate of total days of illness with gastrointestinal tract infections (GTI) in non-elderly adults. Intention-to-treat analysis **A)** assuming no cases among participants with missing data ($I^2 = 90.9$, $p < 0.001$), and **B)** assuming the rate ratio among participants with missing data matched the risk ratio of the control group complete case cohort ($I^2 = 76.7$, $p = 0.001$). Random effects meta-analysis using DerSimonian and Laird inverse variance method. Individual study effect estimates (squares; sized by study weight) and pooled effects (diamonds) are plotted. Lower and upper limits are 95% CIs. Grp, group; L., *Lactobacillus*; NT, non-traveler; Pop., population; T, travelers. Multi-strain_1: *L. acidophilus, L. bulgaricus*; Multi-strain_2: *L. casei* DN-114-001, *Streptococcus thermophilus, L. delbreuckii.* Multi-strain_3: *L. rhamnosus* GG, *B. animalis ssp. lactis* BB12.

Supplemental Figure 5

A

Pop.	Intervention	Study name	Risk ratio	Lower limit	Upper limit	p-Value	GTI / Total -biotic	GTI / Total Placebo
T	GOS	Hasle 2017	0.87	0.62	1.22	0.42	52 / 327	60 / 328
T	GOS	Drakoularakou 2010	0.64	0.39	1.06	0.08	19 / 100	30 / 101
T	FOS	Cummings 2001	0.78	0.56	1.09	0.14	45 / 181	58 / 182
		Pooled	0.79	0.63	0.97	0.03		

Risk ratio and 95% CI

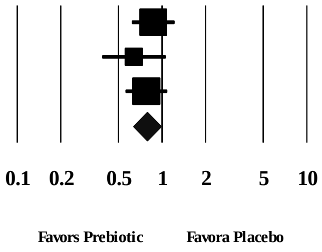

B

Pop.	Intervention	Study name	Risk ratio	Lower limit	Upper limit	p-Value	GTI / Total -biotic	GTI / Total Placebo
T	GOS	Hasle 2017	0.91	0.71	1.16	0.43	88 / 327	97 / 328
T	GOS	Drakoularakou 2010	0.68	0.45	1.03	0.07	26 / 100	39 / 101
T	FOS	Cummings 2001	0.90	0.71	1.14	0.37	74 / 181	83 / 182
		Pooled	0.87	0.74	1.01	0.07		

Risk ratio and 95% CI

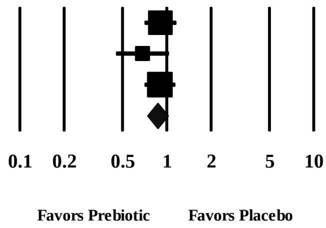

Supplemental Figure 5. Forest plots of the effects of orally ingested prebiotics versus placebo on the risk of experiencing one or more gastrointestinal tract infections (GTI) in non-elderly adults. Intention-to-treat analysis **A)** assuming no cases among participants with missing data ($I^2 = 0$, p = 0.61), and **B)** assuming the risk ratio among participants with missing data matched the risk ratio of the control group complete case cohort ($I^2 = 0$, p = 0.47). Random effects meta-analysis using DerSimonian and Laird inverse variance method. Individual study effect estimates (squares; sized by study weight) and pooled effects (diamonds) are plotted. Lower and upper limits are 95% CIs. FOS, fructooligosaccharide; GOS, galactooligosaccharide; Pop., population; T, traveler.

Supplemental Figure 6

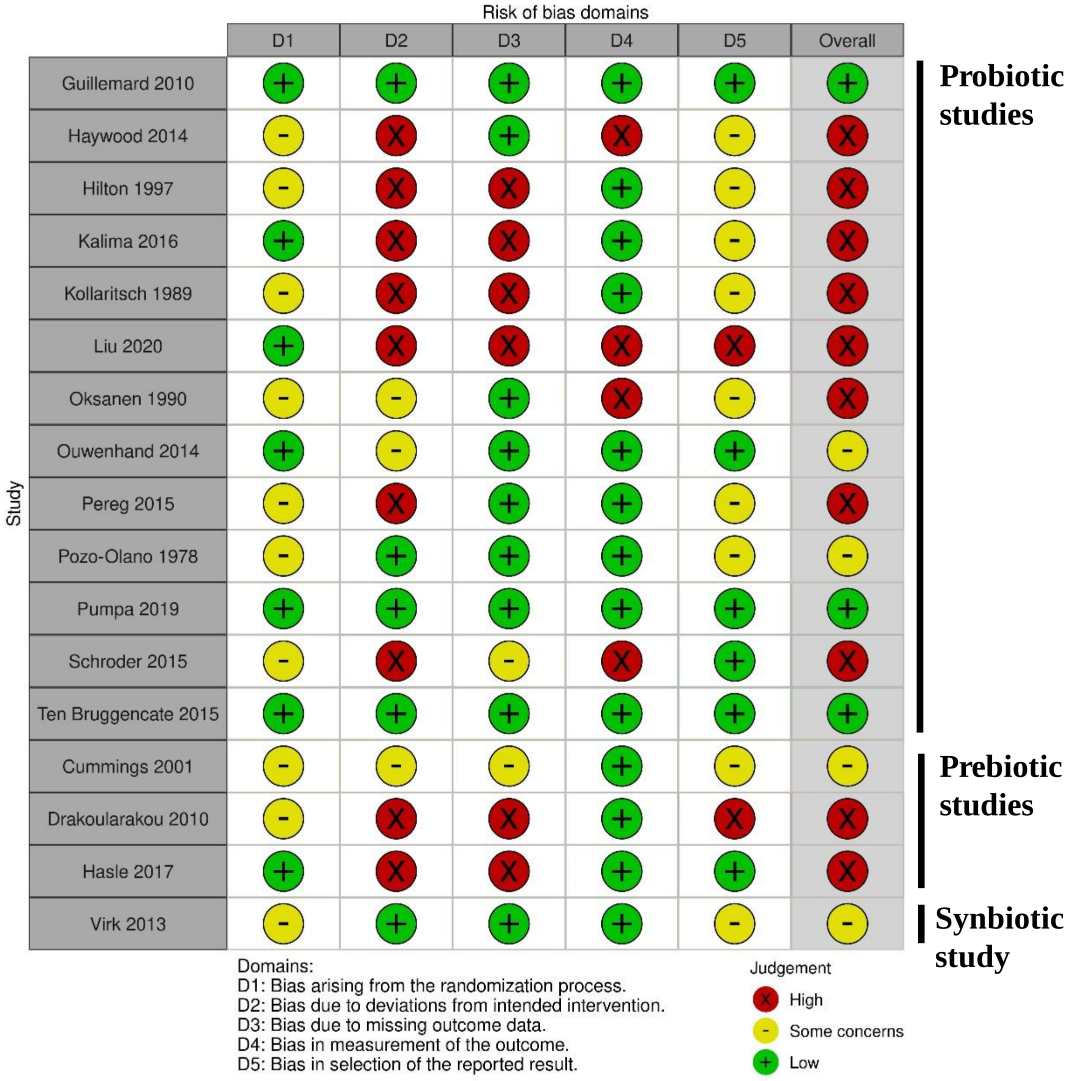

Supplemental Figure 6. Risk of bias assessment for all studies included in systematic review on the effects of probiotics, prebiotics and synbiotics on the incidence, duration and severity of gastrointestinal tract infections in non-elderly adults. Assessed using the Cochrane risk-of-bias assessment tool version 2.0. Plot produced using *robvis* (McGuinness LA and Higgins, JPT. Risk-of-bias VISualization (robvis): An R package and Shiny web app for visualizing risk-of-bias assessments. Res Syn Meth. 2020; 1-7; https://mcguinlu.shinyapps.io/robvis/).

Supplemental Figure 7

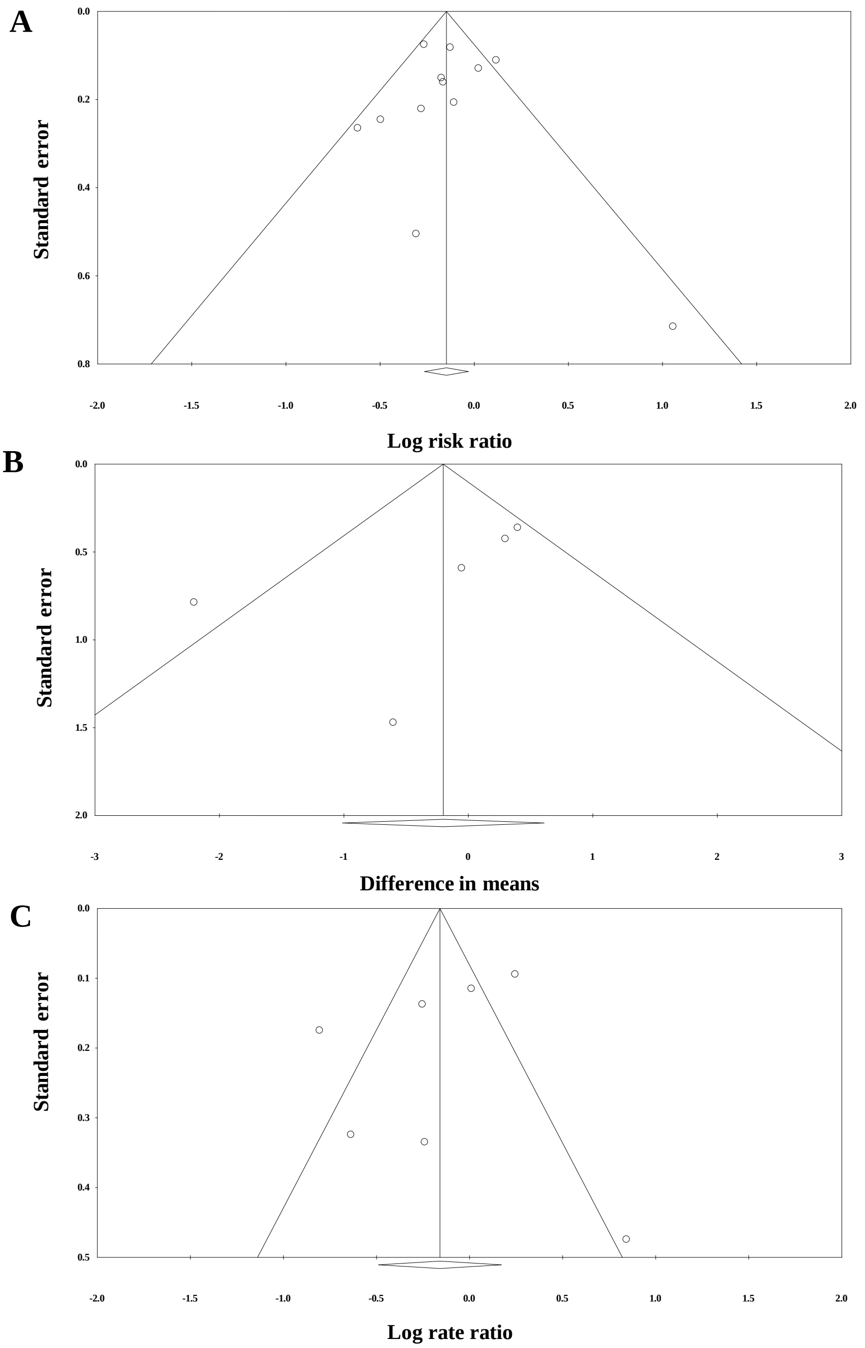

Supplemental Figure 7. Funnel plots for **A)** complete case analyses of effects of probiotics, prebiotics and synbiotics on the risk of experiencing one or more gastrointestinal tract infections (GTI) in non-elderly adults; **B)** complete case analysis of effects of probiotics on the duration of GTI episodes (days/episodes) in non-elderly adults; **C)** complete case analysis of effects of probiotics on the total days of illness from GTI in non-elderly adults.
